# Long-term impact of COVID-19 hospitalisation among individuals with pre-existing airway diseases in the UK: a multicentre, longitudinal cohort study – PHOSP-COVID

**DOI:** 10.1183/23120541.00982-2023

**Published:** 2024-07-15

**Authors:** Omer Elneima, John R. Hurst, Carlos Echevarria, Jennifer K. Quint, Samantha Walker, Salman Siddiqui, Petr Novotny, Paul E. Pfeffer, Jeremy S. Brown, Manu Shankar-Hari, Hamish J.C. McAuley, Olivia C. Leavy, Aarti Shikotra, Amisha Singapuri, Marco Sereno, Matthew Richardson, Ruth M. Saunders, Victoria C. Harris, Linzy Houchen-Wolloff, Neil J. Greening, Ewen M. Harrison, Annemarie B. Docherty, Nazir I. Lone, James D. Chalmers, Ling-Pei Ho, Alex Horsley, Michael Marks, Krisnah Poinasamy, Betty Raman, Rachael A. Evans, Louise V. Wain, Aziz Sheikh, Chris E. Brightling, Anthony De Soyza, Liam G. Heaney

**Affiliations:** 1 The Institute for Lung Health, NIHR Leicester Biomedical Research Centre – Respiratory, University of Leicester, Leicester, UK; 2 UCL Respiratory, Department of Medicine, University College London, London, UK; 3 Royal Free London NHS Foundation Trust, London, UK; 4 Newcastle Upon Tyne Hospitals NHS Foundation Trust, Newcastle Upon Tyne, UK; 5 Translational and Clinical Research Institute, Newcastle University, Newcastle Upon Tyne, UK; 6 School of Public Health, Imperial College London, London, UK; 7 Asthma+Lung UK, London, UK; 8 National Heart and Lung Institute, Imperial College London, London, UK; 9 Department of Respiratory Medicine, Barts Health NHS Trust, London, UK; 10 Barts and The London School of Medicine and Dentistry, Queen Mary University of London, London, UK; 11 Centre for Inflammation Research, University of Edinburgh, Edinburgh, UK; 12 Department of Population Health Sciences, University of Leicester, Leicester, UK; 13 Centre for Exercise and Rehabilitation Science, NIHR Leicester Biomedical Research Centre-Respiratory, University of Leicester, Leicester, UK; 14 Centre for Medical Informatics, The Usher Institute, University of Edinburgh, Edinburgh, UK; 15 University of Dundee, Ninewells Hospital and Medical School, Dundee, UK; 16 MRC Translational Discovery Immunology Unit, University of Oxford, Oxford, UK; 17 NIHR Oxford BRC, Oxford University Hospitals NHS Foundation Trust, Oxford, UK; 18 Division of Infection, Immunity and Respiratory Medicine, Faculty of Biology, Medicine and Health, University of Manchester, Manchester, UK; 19 Manchester University NHS Foundation Trust, Manchester, UK; 20 Department of Clinical Research, London School of Hygiene and Tropical Medicine, London, UK; 21 Hospital for Tropical Diseases, University College London Hospital, London, UK; 22 Division of Cardiovascular Medicine, Radcliffe Department of Medicine, University of Oxford, Oxford, UK; 23 Population Health Sciences Institute, Newcastle University, Newcastle Upon Tyne, UK; 24 Wellcome-Wolfson Institute for Experimental Medicine, Queens University Belfast, Belfast, UK; 25 Belfast Health and Social Care Trust, Belfast, UK; 26 Joint senior authors

## Abstract

**Background:**

The long-term outcomes of COVID-19 hospitalisation in individuals with pre-existing airway diseases are unknown.

**Methods:**

Adult participants hospitalised for confirmed or clinically suspected COVID-19 and discharged between 5 March 2020 and 31 March 2021 were recruited to the Post-hospitalisation COVID-19 (PHOSP-COVID) study. Participants attended research visits at 5 months and 1 year post discharge. Clinical characteristics, perceived recovery, burden of symptoms and health-related quality of life (HRQoL) of individuals with pre-existing airway disease (*i.e.*, asthma, COPD or bronchiectasis) were compared to the non-airways group.

**Results:**

A total of 615 out of 2697 (22.8%) participants had a history of pre-existing airway diseases (72.0% diagnosed with asthma, 22.9% COPD and 5.1% bronchiectasis). At 1 year, the airways group participants were less likely to feel fully recovered (20.4% *versus* 33.2%, p<0.001), had higher burden of anxiety (29.1% *versus* 22.0%, p=0.002), depression (31.2% *versus* 24.7%, p=0.006), higher percentage of impaired mobility using short physical performance battery ≤10 (57.4% *versus* 45.2%, p<0.001) and 27% had a new disability (assessed by the Washington Group Short Set on Functioning) *versus* 16.6%, p=0.014. HRQoL assessed using EQ-5D-5L Utility Index was lower in the airways group (mean±SD 0.64±0.27 *versus* 0.73±0.25, p<0.001). Burden of breathlessness, fatigue and cough measured using a study-specific tool was higher in the airways group.

**Conclusion:**

Individuals with pre-existing airway diseases hospitalised due to COVID-19 were less likely to feel fully recovered, had lower physiological performance measurements, more burden of symptoms and reduced HRQoL up to 1 year post-hospital discharge.

## Shareable abstract

**Following hospitalisation with COVID-19, individuals with pre-existing airway diseases were less likely to feel fully recovered, had more burden of symptoms and reduced health-related quality of life compared to those without pre-existing airway diseases** https://bit.ly/3VfUhcM

## Introduction

Early in the COVID-19 pandemic, the prevalence of asthma and COPD in hospitalised patients with COVID-19 was low, raising the possibility that pre-existing airway diseases or inhaled corticosteroid (ICS) therapy might play a protective role against contracting SARS-CoV-2 infection or severe outcomes [1, 2]. However, later reports found no evidence to support these theories [3, 4], and the number of hospitalised patients with pre-existing airway diseases increased, likely due to relaxation in social distancing [5]. Patients with COPD who were hospitalised were at increased risk of severe COVID-19 illness or death, likely due to factors such as older age, increased number of comorbidities and reduced physiological reserve to survive critical illness [6–9]. Worse clinical outcomes in hospitalised patients with pre-existing asthma were mainly observed in those with severe asthma or those who required multiple courses of oral corticosteroids in the preceding year [10, 11]. Results from a large UK hospitalised cohort found that patients with asthma were more likely to receive critical care than those without asthma [12]. Little is known about the impact of SARS-CoV-2 in patients with pre-existing bronchiectasis largely due to the scarce literature, but there is a suggestion that individuals with pre-existing bronchiectasis had increased risk of worse clinical outcomes after COVID-19 infection [13, 14].

The long-term sequelae following COVID-19 hospitalisation in individuals with pre-existing airway diseases are unknown. An international consensus exercise to determine research priorities in patients with pre-existing airway diseases following COVID-19 hospitalisation identified the need to determine the short- and medium-term effects of COVID-19 infection in this group [15]. Here we report on the results from a large UK-based multicentre cohort study of hospitalised COVID-19 survivors (Post-hospitalisation COVID-19 study (PHOSP-COVID) study).

## Materials and methods

### Study design and participants

The PHOSP-COVID is a UK national multicentre prospective longitudinal cohort study. The PHOSP-COVID study methods have been described in detail elsewhere [16]. Participants were invited to attend two research visits: the first visit between 2 and 7 months; and the second visit between 10 and 14 months post-hospital discharge. Participants were included in the airways group if they self-reported a history of asthma, COPD or bronchiectasis prior to their initial hospitalisation with COVID-19. A small number of the participants indicated a history of combined pre-existing asthma and COPD or bronchiectasis and COPD. Clinical characteristics of this group revealed significant smoking history and older mean age; therefore, these individuals were assigned to the COPD group. Written informed consent was obtained from all participants. The study was approved by the Leeds West Research Ethics Committee (20/YH/0225) and registered on the ISRCTN Registry (ISRCTN10980107).

### Data collection and procedures

Details about the participants' hospital admission were retrospectively collected from the medical records. Research data collected from the two research visits included: patient reported outcome measures (PROMs), health-related quality of life (HRQoL) questionnaires, physiological assessments, routine and research sampling, and pulmonary function tests depending on the local arrangement for aerosol-generating procedures (see supplementary material SM1). HRQoL was measured using EQ-5D-5L Utility Index (UI) and EQ-5D-5L Visual Analogue Scale (VAS).

The Patient Symptom Questionnaire (PSQ) was a study-specific tool used to assess the participants’ perceived full recovery by asking them to answer the question “Do you feel fully recovered from COVID-19?” using the options “Yes”, “No” or “Not sure” at each research visit. The PSQ also assessed the burden of breathlessness, cough, fatigue, sleep disturbance and pain symptoms using a numerical scale ranging from 0 to 10, where 10 represents the highest burden of the symptom. The participants were asked to provide pre-COVID estimates of EQ-5D-5L UI, EQ-5D-5L VAS and burden of symptoms using the PSQ scale.

### Statistical analysis

Descriptive statistics were used to describe participant characteristics. Continuous variables are presented as mean±SD, or medians and interquartile ranges, as appropriate. Binary and categorical variables are presented as counts and percentages of available data. No imputation was performed for the missing data. Results were not adjusted for multiple testing. t-test, analysis of variance (ANOVA F-test) and Kruskal–Wallis H-test were used to compare parametric and non-parametric continuous data as appropriate. Chi-squared test was used to compare categorical data. We did not adjust for cofounders. To examine the predictors of recovery at the second research visit, the participants with pre-existing airway diseases were dichotomised into: “recovered” group for those who answered “Yes” to the perceived full recovery question or “not recovered” group including those who answered “No” or “Not sure” using the PSQ tool. Univariable and multivariable logistic regression were reported to identify predictors of recovery. Only explanatory variables available at hospital discharge were used in the multivariable logistic model comprising: age as a factor, sex at birth, ethnicity, Index of Multiple Deprivation, body mass index (BMI), number of comorbidities, admission duration, severity of acute illness using World Health Organization (WHO) Clinical Progression Scale, history of pre-existing neuropsychiatric disease and the use of systemic steroids during acute admission. R (version 3.6.3) and Stata (version 16.0) were used for all data analysis.

## Results

**FIGURE 1:**
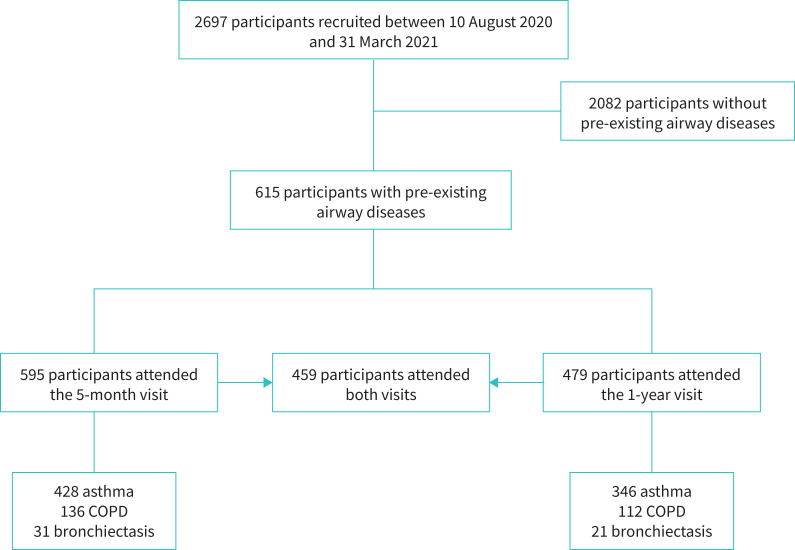
Flow diagram of the participants.

Between 10 August 2020 and 31 March 2022, 2697 participants were recruited to the PHOSP-COVID study and attended at least one research visit. A total of 615 (22.8%) reported a history of pre-existing airway diseases prior to COVID-19 hospitalisation (figure 1, table 1). This included 443 (72.0%) who had a history of asthma, 141 (22.9%) with COPD and 31 (5.1%) with bronchiectasis.

**TABLE 1:** Patient characteristic

	n	Pre-existing airway disease^#^	n	No pre-existing airway disease^¶^	p-value
**Age years**	615	58.7±12.9	2081	57.8±12.5	0.148
**Sex at birth**	615		2081		0.000
** **Male		317 (51.5)		1341 (64.4)	
** **Female		298 (48.5)		740 (35.6)	
**Ethnicity**	608		2070		0.000
** **White		499 (82.1)		1508 (72.9)	
** **South Asian		54 (8.9)		251 (12.1)	
** **Black		33 (5.4)		160 (7.7)	
** **Mixed		10 (1.6)		45 (2.2)	
** **Other		12 (2.0)		106 (5.1)	
**Smoking**	539		1774		0.002
** **Current smoker		23 (4.3)		55 (3.1)	
** **Ex-smoker		234 (43.4)		641 (36.1)	
** **Nonsmoker		282 (52.3)		1078 (60.8)	
**Index of multiple deprivation index (IMD)**	612		2065		0.018
** **1 – most deprived		162 (26.5)		456 (22.1)	
** **2		147 (24.0)		475 (23.0)	
** **3		95 (15.5)		368 (17.8)	
** **4		86 (14.1)		386 (18.7)	
** **5 – least deprived		122 (19.9)		380 (18.4)	
**BMI kg·m^−2^**	432		1441		
** **Median (IQR)		31.9 (28.2–37.4)		30.9 (27.5–35.3)	0.001
** **<30 kg·m^−2^		154 (35.7)		641 (44.6)	
** **≥30 kg·m^−2^		278 (64.3)		796 (55.4)	
**Comorbidities** ^+^	65		2082		
** **Median (IQR)		2 (1–4)		1 (0–3)	0.000
** **0		138 (22.4)		667 (32.0)	0.000
** **1		104 (16.9)		433 (20.8)	
** **≥2		373 (60.7)		982 (47.2)	
** **Cardiovascular	615	297 (48.3)	2082	942 (45.2)	0.183
** **Type 2 diabetes	614	121 (19.7)	2077	416 (20.0)	0.972
** **Neuropsychiatric	615	184 (29.9)	2082	378 (18.2)	0.000
** **Renal and endocrine	615	85 (13.8)	2082	202 (9.7)	0.004
**Hospital admission details**					
** **Admission duration days	615	13.7±16.8	2082	14.2±18.3	0.540
** **Positive SARS-CoV-2 PCR	570	535 (93.9)	1887	1748 (92.6)	0.317
** **WHO clinical progression scale	615		2082		0.041
** **WHO class 3–4		101 (16.4)		346 (16.6)	
** **WHO class 5		278 (45.2)		857 (41.2)	
** **WHO class 6		149 (24.2)		484 (23.2)	
** **WHO class 7–9		87 (14.2)		395 (19.0)	
** **Systemic steroids	583	369 (63.3)	1978	1079 (54.6)	0.000
** **Antibiotic therapy	602	484 (80.4)	2030	1591 (78.4)	0.286
** **Anticoagulants	579	248 (42.8)	1986	925 (46.6)	0.112

Data are presented as n (%), mean±sd or median (IQR). Percentages are calculated by category after exclusion of missing data for that variable. World Health Organization (WHO) classes are as follows: 3–4=no continuous supplemental oxygen needed; 5=continuous supplemental oxygen only; 6=continuous or bi-level positive airway pressure ventilation or high-flow nasal oxygen; and 7–9=invasive mechanical ventilation or other organ support. BMI: body mass index; SARS-CoV-2 PCR: severe acute respiratory syndrome coronavirus 2 polymerase chain reaction. ^#^: n=615; ^¶^: n=2082; ^+^: the total number of comorbidities in the airways group does not include the airway diseases.

Comparison of the participants’ characteristics showed the airways group to have: more females (48.5% *versus* 35.6%, p<0.001), more from a White ethnic background (82.1% *versus* 72.9%, p<0.001), a higher prevalence of pre-existing neuropsychiatric comorbidity (29.9% *versus* 18.2%, p<0.001), higher BMI (median 31.9 *versus* 30.9 kg·m^−2^, p=0.001) and more likely to have received systemic steroids during hospital admission (63.3% *versus* 54.6%, p=0.001) compared to the non-airways group. There was no difference in age, length of hospital admission or treatment with antibiotics or anticoagulants between the two groups. The level of organ support during acute admission was comparable between the two groups with the exception that receiving invasive mechanical ventilation and other organ support (WHO class 7–9) was lower in the airways group (10.0% *versus* 14.2%, p=0.041). At hospital discharge, 63.9% of the airways group were prescribed a form of ICS therapy and 27.2% were on antidepressant medications compared to 1.9% and 16.9% in the non-airways group, respectively. The breakdown of the different classes of prescribed medications upon discharge and additional reported changes at 5-month and 1-year visits are listed in supplementary table S1.

### Results from the 5-month visit

The first research visit was attended by 2570 participants at a median of 5.5 months (IQR 4.1–6.4) from hospital discharge, labelled here as “5-month” visit. A total of 595 out of 2570 (23.2%) participants reported a history of pre-existing airway disease prior to hospital admission (table 2). Assessments at the 5-month visit revealed that the airways group participants were more likely to have symptoms consistent with anxiety (34.4% *versus* 22.8%, p<0.001), depression (44.2% *versus* 26.5%, p<0.001), post-traumatic stress disorder (PTSD) (19.4% *versus* 11.6%, p<0.001) and greater breathlessness measured using the Dyspnoea-12 questionnaire (mean±sd 10.2±9.7 *versus* 5.3±7.4, p<0.001). The airways group participants had a higher percentage of impaired mobility measured using short physical performance battery ≤10 (59.6% *versus* 48.5%, p<0.001) and a lower percentage of predicted incremental shuttle walk test distance (52.4% *versus* 58.7%, p=0.001). They were more likely to be frail using the Rockwood Clinical Frailty score without features of cognitive impairment. Pulmonary function tests revealed lower spirometry measurements in the airways group but no difference in gas transfer measurements between the two groups. The airways group had higher levels of blood neutrophils, eosinophils and higher numbers with systemic inflammation measured by C-reactive protein (CRP) of more than 5 mg·L^−1^ (table 2).

**TABLE 2:** Patient characteristics at the 5-month and 1-year research visits stratified by the presence of pre-existing airway diseases

	5-month visit					1-year visit				
	n	Pre-existing airway disease^#^	n	No pre-existing airway disease^¶^	p-value	n	Pre-existing airway disease^+^	n	No pre-existing airway disease^§^	p-value
**PROMS**										
** **GAD-7 total score	552	6.7±6.1	1856	4.9±5.5	0.000	447	5.9±6.0	1503	4.8±5.5	0.001
** **Anxiety (GAD-7 >8)	552	190 (34.4)	1856	424 (22.8)	0.000	447	130 (29.1)	1503	331 (22.0)	0.002
** **PHQ-9 total score	550	9.0±6.9	1856	6.5±6.3	0.000	443	7.4±6.6	1504	6.1±6.3	0.000
** **Depression (PHQ-9 ≥10)	550	243 (44.2)	1856	491 (26.5)	0.000	443	138 (31.2)	1504	371 (24.7)	0.006
** **PCL-5 total score	552	20.2±18.9	1851	14.6±16.5	0.000	440	17.5±18.4	1497	13.3±16.2	0.000
** **PTSD (PCL-5 ≥38)	552	107 (19.4)	1851	214 (11.6)	0.000	440	74 (16.8)	1497	147 (9.8)	0.000
** **Dyspnoea-12	540	10.2±9.7	1821	5.3±7.4	0.000	430	8.5±8.8	1462	4.9±7.2	0.000
** **FACIT fatigue subscale score	535	29.7±13.6	1791	36.1±12.6	0.000	402	32.7±13.2	1400	36.7±12.4	0.000
**Physical performance**										
** **SPPB total score	539	9.2±2.7	1803	10.0±2.2	0.000	397	9.4±2.5	1397	10.2±2.1	0.000
** **SPPB ≤10 (impaired mobility)	539	321 (59.6)	1803	875 (48.5)	0.000	397	228 (57.4)	1397	632 (45.2)	0.000
** **ISWT distance m	422	364±249	1467	441±267	0.000	295	393±257	1104	456±267	0.000
** **ISWT % predicted	305	52.4±30.3	1032	58.7±30.0	0.001	226	54.0±30.8	796	60.8±30.3	0.003
**Frailty and cognition**										
** **Rockwood CFS score ≥5	541	65 (12.0)	1743	70 (4.0)	0.000	422	45 (10.7)	1463	59 (4.0)	0.000
** **SARC-F total score	541	2.8±2.5	1785	1.7±2.1	0.000	403	2.5±2.5	1405	1.7±2.1	0.000
** **MoCA total score	482	25.4±3.8	1616	25.7±3.4	0.074	379	26.3±3.3	1303	26.3±3.4	0.708
** **Corrected MoCA total score	482	25.8±3.8	1616	26.1±3.4	0.126	379	26.7±3.2	1303	26.6±3.3	0.443
** **MoCA <23	482	85 (17.6)	1616	236 (14.6)	0.105	379	47 (12.4)	1303	152 (11.7)	0.696
** **Corrected MoCA <23	482	74 (15.4)	1616	205 (12.7)	0.130	379	39 (10.3)	1303	139 (10.7)	0.833
**Lung physiology**										
** **FEV_1_ L	366	2.47±0.77	1150	2.85±0.79	0.000	241	2.52±0.81	840	2.89±0.80	0.000
** **FEV_1_ % predicted	341	84.5±19.4	1098	91.7±17.8	0.000	231	86.5±21.2	820	93.2±17.4	0.000
** **FEV_1_ % predicted <80%	341	135 (39.6)	1098	255 (23.2)	0.000	231	84 (36.4)	820	173 (21.1)	0.000
** **FVC L	366	3.24±0.94	1150	3.55±1.03	0.000	241	3.33±0.98	840	3.61±1.01	0.000
** **FVC % predicted	341	87.2±17.3	1098	89.7±19.0	0.029	231	89.7±20.4	820	91.2±17.8	0.289
** **FVC % predicted <80%	341	116 (34.0)	1098	311 (28.3)	0.043	231	78 (33.8)	820	188 (22.8)	0.001
** **FEV_1_/FVC	366	0.77±0.12	1150	0.81±0.09	0.000	241	0.77±0.19	840	0.81±0.11	0.000
** **FEV_1_/FVC <0.7	341	80 (23.5)	1098	83 (7.6)	0.000	241	55 (22.8)	840	63 (7.5)	0.000
** ** *T*_LCO_	122	7.08±2.13	389	7.53±2.38	0.062	76	7.62±2.33	264	7.69±2.36	0.832
** ***T*_LCO_ % predicted	121	90.7±26.8	378	91.9±32.5	0.718	76	96.8±28.1	261	95.0±29.7	0.628
** ***T*_LCO_ % predicted <80%	121	45 (37.2)	378	130 (34.4)	0.574	76	18 (23.7)	261	60 (23.0)	0.899
** ** *K*_CO_	127	1.49±0.28)	393	1.45±0.32)	0.214	80	1.46±0.28)	273	1.44±0.27)	0.454
** ***K*_CO_ % predicted	127	102.3±18.0	380	100.5±20.9	0.387	80	101.9±18.2	270	100.0±17.3	0.405
** ***K*_CO_ % predicted <80%	127	9 (7.1)	380	36 (9.5)	0.413	80	9 (11.3)	270	24 (8.9)	0.526
**Biochemical tests**										
** **Haemoglobin	501	139.6±15.0	1640	141.5±15.4	0.012	383	139.6±15.7	1280	141.6±14.8	0.024
** **Neutrophils	500	4.5±1.8	1635	4.0±1.5	0.000	381	4.6±2.2	1275	4.0±1.5	0.000
** **Eosinophils	495	0.23±0.22	1626	0.18±0.17	0.000	381	0.22±0.18	1275	0.19±0.18	0.001
** **BNP/Pro-NT-BNP above threshold	378	25 (6.6)	1197	76 (6.4)	0.867	237	23 (9.7)	824	68 (8.3)	0.482
** **HbA1C ≥6.0	394	150 (38.1)	1236	427 (34.6)	0.203	279	107 (38.4)	1008	355 (35.2)	0.334
** **eGFR <60 (mL/min/1.73 m^2^)	488	57 (11.7)	1581	155 (9.8)	0.232	358	46 (12.9)	1227	153 (12.5)	0.853
**Systemic inflammation**										
** **CRP mg·L^−1^	472	6.2±9.4	1580	5.2±11.2	0.074	374	5.8±6.4	1260	4.9±7.0	0.021
** **CRP >5 mg·L^−1^	472	146 (30.9)	1580	348 (22.0)	0.000	374	114 (30.5)	1260	278 (22.1)	0.001
** **CRP ≥10 mg·L^−1^	472	73 (15.5)	1580	155 (9.8)	0.001	374	53 (14.2)	1260	120 (9.5)	0.011

Data are presented as n (%) or mean±sd. Percentages are calculated by category after exclusion of missing data for that variable. Threshold of BNP ≥100 ng·L^−1^ or NT-BNP ≥400 ng·L^−1^. Corrected MoCA adjusted for level of education. One was subtracted from the total number of comorbidities in the airways group. See Table SM1 for further descriptions of variables. PROMs: patient reported outcome measures; GAD7: Generalised Anxiety Disorder 7-item scale; PHQ-9: Patient Health Questionnaire-9; PCL-5: Post-Traumatic Stress Disorder (PTSD) Checklist; FACIT fatigue: Functional Assessment of Chronic Illness Therapy Fatigue Scale; SPPB: short physical performance battery; ISWT: incremental shuttle walk test; CFS: Clinical Frailty Scale; MoCA: Montreal Cognitive Assessment; FEV_1_: forced expiratory volume in 1 s; FVC: forced vital capacity; *T*_LCO_: transfer capacity of the lung for carbon monoxide; *K*_CO_: carbon monoxide transfer coefficient; BNP: brain natriuretic peptide; NT-BNP: N-terminal BNP; HbA1C: glycated haemoglobin; eGFR: estimated glomerular filtration rate; CRP: C-reactive protein. ^#^: n=595; ^¶^: n=1975; ^+^: n=479; ^§^: n=1621.

**FIGURE 2:**
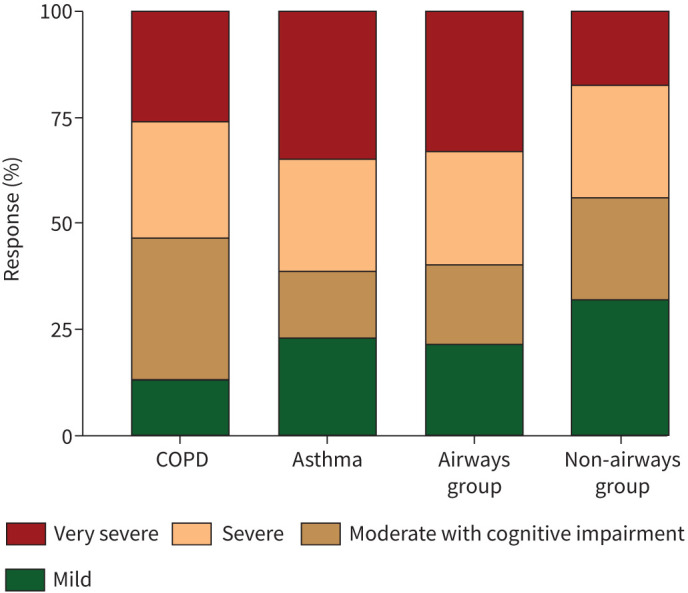
Recovery cluster membership assignment at 5-month visit stratified by the underlying class of airway disease. The four clusters are: very severe mental and physical impairment, severe mental and physical impairment, moderate mental and physical impairment with cognitive impairment, and mild.

The perceived recovery question demonstrated a lower proportion of participants in the airways group reporting “full recovery” (19.7% *versus* 27.6%, p=0.005) (table 3). The participants who attended the 5-month visit were assigned one of the previously identified four cluster memberships of recovery phenotypes [17]: very severe, severe, moderate with cognitive impairment, and mild mental and physical impairment (see supplementary material SM1). Recovery cluster assignment was different between the airways and non-airways groups with a higher proportion of those with pre-existing airway diseases assigned to the very severe mental and physical impairment cluster (32.5% *versus* 17.5%) and a smaller proportion assigned to the mild cluster (21.1% *versus* 32.7%), p<0.001 (figure 2, supplementary table S2).

**TABLE 3:** Recovery, health-related quality of life and symptoms burden at the 5-month and 1-year research visits stratified by the presence of pre-existing airway diseases

	5-month visit					1-year visit				
	n	Pre-existing airway disease^#^	n	No pre-existing airway disease^¶^	p-value	n	Pre-existing airway disease^+^	n	No pre-existing airway disease^§^	p-value
**Fully recovered from COVID-19?**	509		1693		0.002	403		1384		0.000
** **Yes		100 (19.7)		467 (27.6)			82 (20.4)		459 (33.2)	
** **No		303 (59.5)		912 (53.9)			225 (55.8)		638 (46.1)	
** **Not sure		106 (20.8)		314 (18.5)			96 (23.8)		287 (20.7)	
**EQ-5D-5L Utility Index pre-COVID estimate**	503	0.74±0.27	1667	0.84±0.21	0.000	422	0.74±0.27	1399	0.84±0.21	0.000
**EQ-5D-5L Utility Index at the visit**	487	0.62±0.28	1626	0.73±0.24	0.000	390	0.64±0.27	1350	0.73±0.25	0.000
**EQ-5D-5L Utility Index delta change**	404	−0.12±0.26	1353	−0.11±0.21	0.451	338	−0.10±0.24	1160	−0.11±0.22	0.351
**EQ5D-5L VAS pre-COVID estimate**	491	73.7±18.5	1604	81.3±16.8	0.000	410	74.6±18.9	1342	81.1±16.6	0.000
**EQ5D-5L VAS at the visit**	488	63.4±21.3	1618	72.2±19.2	0.000	384	67.3±21.2	1347	71.3±20.4	0.001
**EQ5D-5L VAS delta change**	397	−10.6±21.3	1300	−9.7±18.8	0.441	323	−8.5±20.2	1112	−10.2±19.7	0.168
**WG-SS-SCo**	508	176±34.7	1700	356±20.9	0.000	407	130±31.9	1386	259±18.7	0.000
**WG-SS-SCo new disability**	384	94 (24.5)	1275	223 (17.5)	0.002	111	30 (27.0)	380	63 (16.6)	0.014
**PSQ Breathlessness pre-COVID estimate**	493	2.6±2.6	1669	0.8±1.7	0.000	256	2.6±2.4	837	0.8±1.6	0.000
**PSQ Breathlessness at the visit**	496	4.9±2.8	1697	3.7±2.9	0.000	398	3.7±2.7	1372	2.4±2.5	0.000
**PSQ Breathlessness delta change**	481	2.3±3.1	1628	2.9±3.0	0.000	243	1.2±2.7	805	1.9±2.7	0.000
**PSQ Cough pre-COVID estimate**	489	1.7±2.4	1664	0.6±1.5	0.000	256	1.5±2.2	836	0.6±1.5	0.000
**PSQ Cough at the visit**	491	2.9±2.9	1693	1.9±2.6	0.000	395	2.1±2.5	1368	1.3±2.1	0.000
**PSQ Cough delta change**	476	1.3±3.0	1622	1.3±2.7	0.643	241	0.6±2.4	801	0.9±2.3	0.100
**PSQ Fatigue pre-COVID estimate**	488	2.2±2.6	1664	1.4±2.2	0.000	256	2.1±2.4	831	1.3±2.0	0.000
**PSQ Fatigue at the visit**	490	5.7±2.9	1693	4.5±3.0	0.000	396	4.3±3.0	1369	3.3±2.9	0.000
**PSQ Fatigue delta change**	474	3.5±3.3	1621	3.1±3.2	0.060	243	2.5±2.0	799	2.4±2.2	0.979
**PSQ Sleep Disturbance pre-COVID estimate**	488	2.8±2.7	1663	1.9±2.5	0.000	255	2.5±2.7	836	1.8±2.4	0.000
**PSQ Sleep Disturbance at the visit**	491	4.9±3.0	1686	3.8±3.1	0.000	398	3.9±3.1	1368	3.3±3.0	0.000
**PSQ Sleep Disturbance delta change**	473	2.1±3.0	1616	1.9±3.1	0.136	243	1.5±2.9	805	1.6±2.9	0.739
**PSQ Pain pre-COVID estimate**	489	2.3±2.9	1649	1.4±2.4	0.000	253	2.3±2.8	836	1.4±2.3	0.000
**PSQ Pain at the visit**	492	4.0±3.4	1677	3.0±3.1	0.000	396	3.3±3.1	1363	2.5±2.8	0.000
**PSQ Pain delta change**	477	1.7±2.9	1601	1.6±2.8	0.320	239	1.2±2.7	800	1.3±2.6	0.653

Data are presented as n (%) or mean±sd. Missing not included in %. Delta change at each visit was calculated from the pre-COVID estimates. EQ-5D-5L VAS: Euroqol five level visual analogue scale 0–100; WG-SS-SCo: Washington Group Short Set of Functioning Severity Continuum; PSQ: Patient Symptoms Questionnaires. See supplementary table SM1 for further descriptions of variables. ^#^: n=595; ^¶^: n=1975; ^+^: n=479; ^§^: n=1621.

**FIGURE 3:**
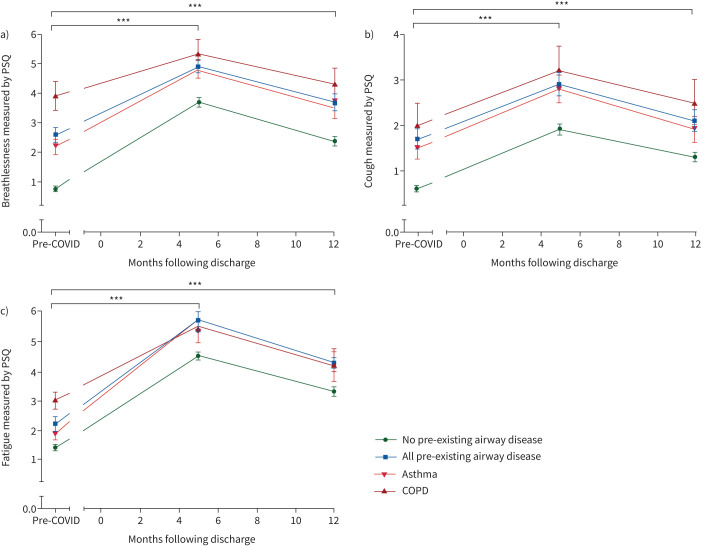
Symptoms over time. Symptoms change measured using Patient Symptoms Questionnaire (PSQ) from pre-COVID, to 5-month and 1-year visits stratified by presence or absence of pre-existing airway diseases: a) breathlessness, b) cough and c) fatigue. ***p<0.001.

HRQoL assessed using EQ-5D-5L UI and EQ-5D-5L VAS showed lower estimated pre-hospitalisation levels in the airways group (0.74±0.27 *versus* 0.84±0.21 and 73.7±18.5 *versus* 81.3±16.8, all p<0.001), respectively. The participants in the airways group reported a drop in the EQ-5D-5L UI of 0.12±0.26 units similar to the non-airways group (0.11±0.21 units, p=0.451). A higher proportion of the airways group reached the threshold for a new disability using the Washington Group Short Set on Functioning (WG-SS), 24.5% *versus* 17.5%, p=0.002. In the airways group, the burden of breathlessness, cough, fatigue, sleep disturbance and pain measured using the PSQ scale was higher both at pre-COVID estimate and at the 5-month visit (table 3, figure 3). However, delta difference between pre-COVID level and the 5-month visit was smaller in the case of breathlessness (2.3 *versus* 2.9, p<0.001) in the airways group compared to the non-airways group but similar in cough, fatigue, sleep disturbance and pain.

**FIGURE 4:**
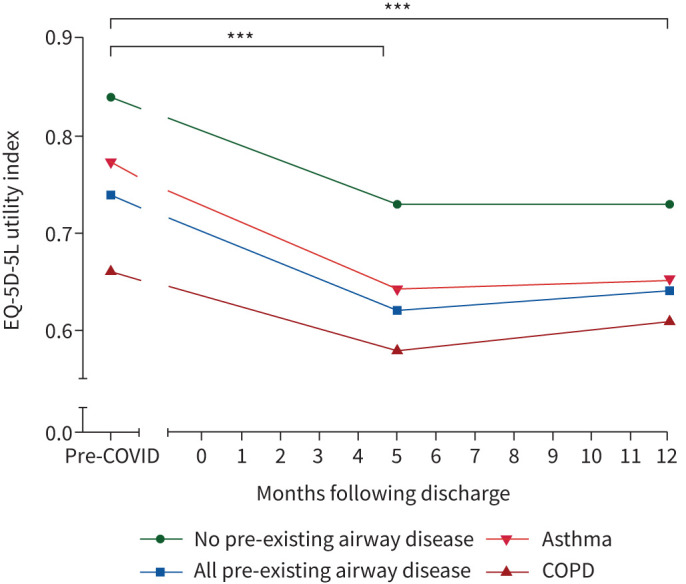
Change in health-related quality of life (HRQoL) measured using EQ-5D-5L Utility Index from pre-COVID to 5-month and 1-year visit by presence or absence of pre-existing airway diseases. COPD: chronic obstructive pulmonary disease. p-values calculated using t-test: ***p<0.001.

The differences in clinical characteristics of the participants attending the 5-month visit stratified by the underlying class of airway disease are included in supplementary tables S3 and S4. The COPD group were older, had more male participants, were mainly from a White background, were less likely to have received invasive ventilation, had more comorbidities and were more likely to be assigned to the moderate mental and physical impairment with cognitive impairment cluster. The COPD group at the 5-month visit were frailer, had a higher percentage of impaired mobility, more evidence of cognitive impairment and showed features of anxiety and depression in over 30% of the group. They also had the lowest spirometry measurements but comparable gas transfer readings. Blood tests revealed higher levels of neutrophils, eosinophils, CRP and higher proportion of participants with heart failure or renal impairment. The COPD group had the smallest drop in EQ-5D-5L UI and VAS measurements despite having the lowest estimates pre-COVID (figure 4). They also had minimal increase in the burden of breathlessness, cough and fatigue measured using PSQ despite having higher levels of baseline burden (figure 3).

The participants with underlying asthma were characterised by: younger age, more females, higher BMI, more likely to report “not recovered” and more than a third were assigned to the very severe cluster. The asthma group had the largest drop in EQ-5D-5L UI and VAS and the highest increase in burden of fatigue (supplementary table S4, figure 3). The change in breathlessness and cough in the asthma group was similar to those without pre-existing airway diseases (figure 3).

### Results from the 1-year visit

A total of 2100 participants attended a second research visit at “1 year” at a median of 12.6 months (IQR 11.8–13.4) from hospital discharge. A total of 479 (22.8%) participants had a history of pre-existing airway diseases (figure 1). At 1-year visit, the airways group participants remained less likely to report full recovery compared to the non-airways group (20.4% *versus* 33.2%, p<0.001) and were more likely to have features consistent with anxiety, depression, PTSD, increased frailty, reduced physical performance and higher CRP levels (table 2). HRQoL measurements at 1 year in the airways group were lower than in the non-airways group and showed no improvement from the 5-month levels with no recovery to pre-COVID estimates (figure 4). A higher proportion of the airways group reached the threshold for a new disability using the WG-SS (27% *versus* 16.6%, p=0.014).

In the airways group there was some improvement in the burden of symptoms between the 5-month and 1-year visit: anxiety (34.4% to 29.1%), depression (44.2% to 31.1%), cognitive impairment (15.4% to 10.3%) and breathlessness measured using Dyspnoea-12 (mean 10.2 to 8.5), table 2. Despite the participants in the airways group having a higher burden of the symptoms measured using PSQ compared to the non-airways group at 1 year, there was a trend towards improvement from the 5-month levels (figure 3). Clinical characteristics of the participants who attended the 1-year visit stratified by the underlying class of airway diseases are available in supplementary tables S5 and S6.

### Factors predicting recovery at 1 year

At the 1-year visit, data about perceived recovery were available in 403 out of 479 (84.1%) of the airways group and 1384 out of 1621 (85.3%) of the non-airways group. The characteristics of the recovered participants in the airways group are listed in supplementary table S7. The multivariable logistic regression model did not identify any statistically significant features to predict recovery at 1 year post discharge in the airways group; however, non-White ethnicity, age ≥70 years and receiving noninvasive respiratory support during initial admission were associated with increased likelihood of recovery (supplementary table S8, figure SF1). In contrast, female sex, history of neuropsychiatric comorbidity, increased level of deprivation, having one or more comorbidity, and receiving invasive ventilation/organ support were suggestive of reduced likelihood of full recovery. These predicting factors were similar in the non-airways group with the features of female, non-White ethnicity and pre-existing neuropsychiatric comorbidity reaching statistical significance (supplementary table S9 and figure SF2).

## Discussion

To our knowledge, this is the first report to focus on the long-term impact of COVID-19 hospitalisation on individuals with pre-existing airway diseases using results from a large multicentre prospective longitudinal UK cohort study. Around a quarter of the PHOSP-COVID cohort had a history of pre-existing airway diseases with the majority of those reporting asthma. Individuals with pre-existing airway diseases were less likely to feel fully recovered at 5-month and at 1-year post-hospital discharge and they had a significant burden of anxiety, depression, PTSD, breathlessness, cough and fatigue compared to the non-airways group. There was evidence of reduced physiological performance, lower spirometry measurements, reduced level of HRQoL both pre-COVID and at the follow-up visits with raised neutrophils, eosinophils and systemic inflammation measured using CRP in the airways group.

Although the prevalence of airway diseases in our cohort might seem high, it is comparable to reports from the large ISARIC study and to the prevalence of asthma in the age group of 55–64 years in the UK [12]. Furthermore, around two-thirds of the airways group were prescribed inhaled bronchodilators or ICS therapy. The results from this cohort study support previous findings from smaller studies including a UK-wide survey where patients with pre-existing lung diseases were more likely to report “breathing complications” after contracting COVID-19 [18]. Another UK-based online survey among patients with underlying asthma revealed that more than half of the participants experienced features of “long COVID” that were not related to personal characteristics such as age, sex, ethnicity or household income [19]. A cohort study of 2649 participants from Russia revealed that patients with chronic pulmonary diseases were more likely to report respiratory symptoms and chronic fatigue [20]. Numerous reports identified breathlessness as one of the commonest persistent symptoms post-COVID in the general population [21–23]. In our study, people with pre-existing airway disease had a higher burden of breathlessness measured using the Dyspnoea-12 questionnaire and PSQ breathlessness scale at both visits. However, the non-airways group had the largest increase in breathlessness from pre-COVID levels measured using the PSQ scale. As expected, spirometry results were lower in the airways group, but we observed no difference in gas transfer measurements between the two groups. Despite the smaller number of participants who completed these procedures (due to the restrictions around aerosol-generating procedures), the latter finding suggests that a pre-existing airway disease is not necessarily a major risk factor for further lung function impairment post-COVID-19 hospitalisation and the pathophysiology of persistent breathlessness is likely to be multifactorial [24].

Other symptoms of cough, sleep disturbance and pain were higher in the airways group at pre-COVID baseline but increased in similar proportion to the non-airways group, resulting in an overall higher burden of these symptoms at both visits in those with pre-existing airway disease. Fatigue is highly prevalent in patients with airway diseases [25, 26], and the COPD group in our cohort had double the level of fatigue burden pre-COVID compared to the non-airways group. Interestingly, the asthma group, which was dominated by female participants, demonstrated the highest increase in fatigue burden compared to the COPD and the non-airways groups. Multiple reports have identified female sex as an independent risk factor for developing chronic fatigue post-COVID-19 [22, 27, 28].

Patients with pre-existing airway diseases are known to have reduced HRQoL compared to controls [29–31]. In our study, HRQoL in the airways group was reduced both at pre-COVID estimates and at both follow-up visits, with minimal improvement between 5 months and 1 year post discharge. This was similar to the results of 1-year follow-up of 1276 COVID-19 survivors from Wuhan, China [21] and a German study of COVID-19 survivors who required intensive care unit admission [32], where HRQoL remained reduced at 1 year after hospital discharge. Interestingly, in our cohort the magnitude of decline in HRQoL from pre-COVID estimates to the research visits was similar in both groups, suggesting that patients with pre-existing airway diseases are not at increased risk of significant deterioration of HRQoL compared to those without airway diseases. There was a general trend of improvement in the burden of symptoms between the 5-month and 1-year visit in the overall cohort and more specifically in the airways group. This was similar to the findings from the Wuhan study [21].

Owing to the small number of the bronchiectasis cases in this cohort, reaching robust conclusions about the long-term sequelae of COVID-19 in this disease is challenging, but our results support earlier indications that pre-existing bronchiectasis is associated with increased morbidity after COVID-19 [13, 14].

The multivariable logistic regression suggested that in both airways and non-airways groups, being female, more severe acute illness, increased number of comorbidities and history of pre-existing neuropsychiatric diseases were all associated with reduced likelihood of reporting full recovery at 1 year post discharge. These identified risk factors were consistent with previously published systematic reviews and meta-analyses exploring the risk factors of prolonged symptoms post-COVID in hospitalised individuals [21, 33–35].

Results from this cohort study are important for policy decisions and clinical practice as they highlight the significant burden of symptoms and morbidity in an already vulnerable group [36]. The challenges facing healthcare providers globally will likely worsen the clinical outcomes of individuals with pre-existing airway diseases unless healthcare provision is prioritised in this group in the form of offering pulmonary rehabilitation, reviews of inhaler technique, delivery of vaccinations, clinical monitoring and self-management plan implementation [37, 38]. The high prevalence of anxiety, depression and PTSD in this group highlights the rising need to improve access to mental health and counselling services [39, 40].

The strengths of this cohort analysis include reporting the findings from a large multicentre study with in-depth assessments using validated and novel tools to measure recovery, burden of symptoms and HRQoL in hospitalised COVID-19 individuals with pre-existing airway diseases. Our study had several limitations. First, the high prevalence of symptoms across the whole of the PHOSP-COVID study participants raises the possibility of selection bias where individuals with high burden of symptoms choose to participate in the study. Second, we relied on the recall of certain measurements by the participants including the history of pre-existing illness and the pre-COVID estimates of HRQoL and burden of symptoms. This includes the use of the study-specific PSQ, which is not externally validated but supports results from other validated tools, *e.g.*, Dyspnoea-12, FACIT fatigue subscale scores. Although a large proportion of the airways group individuals were prescribed inhaled therapy on hospital discharge, which supports the self-reported diagnosis of pre-existing airway disease, this observation alone cannot confirm a pre-existing diagnosis due to discrepancy between prescribing inhaled therapy and the prevalence of diagnosed asthma and COPD [41, 42]. Third, the lack of a control group of participants with pre-existing airway diseases who were not hospitalised for COVID-19 infection is also a limitation; however, the overall design of the PHOSP-COVID study did not include a control group of non-hospitalised individuals. Fourth, it is not clear how much of the reported burden of symptoms up to 1 year post discharge can be attributed to the pre-existing airway diseases *versus* emergent impaired health status. Fifth, no data were collected regarding the frequency or severity of exacerbations of pre-existing airway diseases nor the use of rescue medications. Sixth, the participants in this cohort were mainly individuals who were hospitalised during the first wave of the pandemic in the UK prior to the widespread use of in-hospital COVID-19 therapeutic interventions and the uptake of vaccination, therefore limiting the generalisability of these findings to the overall population.

In conclusion, individuals with pre-existing airway diseases who were hospitalised due to COVID-19 were less likely to feel fully recovered and had greater burden of symptoms and reduced HRQoL up to 1 year post discharge. Prioritisation of clinical care provision in this group is essential to minimise further decline in health status in an already premorbid population.

## Supplementary material

10.1183/23120541.00982-2023.Supp1 **Please note:** supplementary material is not edited by the Editorial Office, and is uploaded as it has been supplied by the author.
Supplementary material 00982-2023.SUPPLEMENT

## References

[1] Halpin DMG, Faner R, Sibila O, et al. Do chronic respiratory diseases or their treatment affect the risk of SARS-CoV-2 infection? Lancet Respir Med 2020; 8: 436–438. doi:10.1016/S2213-2600(20)30167-3 32251625 PMC7270536

[2] Rogliani P, Lauro D, Di Daniele N, et al. Reduced risk of COVID-19 hospitalization in asthmatic and COPD patients: a benefit of inhaled corticosteroids? Expert Rev Respir Med 2021; 15: 561–568. doi:10.1080/17476348.2021.1850275 33183113 PMC7752139

[3] Schultze A, Walker AJ, MacKenna B, et al. Risk of COVID-19-related death among patients with chronic obstructive pulmonary disease or asthma prescribed inhaled corticosteroids: an observational cohort study using the OpenSAFELY platform. Lancet Respir Med 2020; 8: 1106–1120. doi:10.1016/S2213-2600(20)30415-X 32979987 PMC7515601

[4] Halpin DMG, Singh D, Hadfield RM. Inhaled corticosteroids and COVID-19: a systematic review and clinical perspective. Eur Respir J 2020; 55: 2001009. doi:10.1183/13993003.01009-2020 32341100 PMC7236828

[5] Docherty AB, Harrison EM, Green CA, et al. Features of 20 133 UK patients in hospital with covid-19 using the ISARIC WHO Clinical Characterisation Protocol: prospective observational cohort study. BMJ 2020; 369: m1985. doi:10.1136/bmj.m1985 32444460 PMC7243036

[6] Sanchez-Ramirez DC, Mackey D. Underlying respiratory diseases, specifically COPD, and smoking are associated with severe COVID-19 outcomes: a systematic review and meta-analysis. Respir Med 2020; 171: 106096. doi:10.1016/j.rmed.2020.106096 32763754 PMC7391124

[7] Graziani D, Soriano JB, Del Rio-Bermudez C, et al. Characteristics and prognosis of COVID-19 in patients with COPD. J Clin Med 2020; 9: 3259. doi:10.3390/jcm9103259 33053774 PMC7600734

[8] Gerayeli FV, Milne S, Cheung C, et al. COPD and the risk of poor outcomes in COVID-19: a systematic review and meta-analysis. EClinicalMedicine 2021; 33: 100789. doi:10.1016/j.eclinm.2021.100789 33758801 PMC7971471

[9] Aveyard P, Gao M, Lindson N, et al. Association between pre-existing respiratory disease and its treatment, and severe COVID-19: a population cohort study. Lancet Respir Med 2021; 9: 909–923. doi:10.1016/S2213-2600(21)00095-3 33812494 PMC8016404

[10] Williamson EJ, Walker AJ, Bhaskaran K, et al. Factors associated with COVID-19-related death using OpenSAFELY. Nature 2020; 584: 430–436. doi:10.1038/s41586-020-2521-4 32640463 PMC7611074

[11] Adir Y, Saliba W, Beurnier A, et al. Asthma and COVID-19: an update. Eur Respir Rev 2021; 30: 210152. doi:10.1183/16000617.0152-2021 34911694 PMC8674937

[12] Bloom CI, Drake TM, Docherty AB, et al. Risk of adverse outcomes in patients with underlying respiratory conditions admitted to hospital with COVID-19: a national, multicentre prospective cohort study using the ISARIC WHO Clinical Characterisation Protocol UK. Lancet Respir Med 2021; 9: 699–711. doi:10.1016/S2213-2600(21)00013-8 33676593 PMC8241313

[13] Choi H, Lee H, Lee SK, et al. Impact of bronchiectasis on susceptibility to and severity of COVID-19: a nationwide cohort study. Ther Adv Respir Dis 2021; 15: 1753466621995043. doi:10.1177/1753466621995043 33583345 PMC7890711

[14] Guan WJ, Liang WH, Shi Y, et al. Chronic respiratory diseases and the outcomes of COVID-19: a nationwide retrospective cohort study of 39,420 cases. J Allergy Clin Immunol Pract 2021; 9: 2645–2655.e14. doi:10.1016/j.jaip.2021.02.041 33684635 PMC7935669

[15] Adeloye D, Elneima O, Daines L, et al. The long-term sequelae of COVID-19: an international consensus on research priorities for patients with pre-existing and new-onset airways disease. Lancet Respir Med 2021; 9: 1467–1478. doi:10.1016/S2213-2600(21)00286-1 34416191 PMC8372501

[16] Elneima O, McAuley HJC, Leavy OC, et al. Cohort profile: post-hospitalisation COVID-19 study (PHOSP-COVID). Int J Epidemiol 2023; 53: dyad165. doi:10.1093/ije/dyad165 PMC10859139 39429158

[17] Evans RA, McAuley H, Harrison EM, et al. Physical, cognitive, and mental health impacts of COVID-19 after hospitalisation (PHOSP-COVID): a UK multicentre, prospective cohort study. Lancet Respir Med 2021; 9: 1275–1287. doi:10.1016/S2213-2600(21)00383-0 34627560 PMC8497028

[18] Buttery S, Philip KEJ, Williams P, et al. Patient symptoms and experience following COVID-19: results from a UK-wide survey. BMJ Open Respir Res 2021; 8: e001075. doi:10.1136/bmjresp-2021-001075 PMC8572361 34732518

[19] Philip KEJ, Buttery S, Williams P, et al. Impact of COVID-19 on people with asthma: a mixed methods analysis from a UK wide survey. BMJ Open Respir Res 2022; 9: e001056. 10.1136/bmjresp-2021-001056 PMC8762134 35027428

[20] Munblit D, Bobkova P, Spiridonova E, et al. Incidence and risk factors for persistent symptoms in adults previously hospitalized for COVID-19. Clin Exp Allergy 2021; 51: 1107–1120. doi:10.1111/cea.13997 34351016 PMC8444748

[21] Huang L, Yao Q, Gu X, et al. 1-year outcomes in hospital survivors with COVID-19: a longitudinal cohort study. Lancet 2021; 398: 747–758. doi:10.1016/S0140-6736(21)01755-4 34454673 PMC8389999

[22] Sigfrid L, Drake TM, Pauley E, et al. Long Covid in adults discharged from UK hospitals after Covid-19: a prospective, multicentre cohort study using the ISARIC WHO Clinical Characterisation Protocol. Lancet Reg Health Eur 2021; 8: 100186. doi:10.1016/j.lanepe.2021.100186 34386785 PMC8343377

[23] Aiyegbusi OL, Hughes SE, Turner G, et al. Symptoms, complications and management of long COVID: a review. J R Soc Med 2021; 114: 428–442. doi:10.1177/01410768211032850 34265229 PMC8450986

[24] Daines L, Zheng B, Elneima O, et al. Characteristics and risk factors for post-COVID-19 breathlessness after hospitalisation for COVID-19. ERJ Open Res 2023; 9: 00274–0. doi:10.1183/23120541.00274-2022 36820079 PMC9790090

[25] Baghai-Ravary R, Quint JK, Goldring JJ, et al. Determinants and impact of fatigue in patients with chronic obstructive pulmonary disease. Respir Med 2009; 103: 216–223. doi:10.1016/j.rmed.2008.09.022 19027278

[26] Van Herck M, Spruit MA, Burtin C, et al. Fatigue is highly prevalent in patients with asthma and contributes to the burden of disease. J Clin Med 2018; 7: 471. doi:10.3390/jcm7120471 30477110 PMC6306949

[27] Rudroff T, Workman CD, Bryant AD. Potential factors that contribute to post-COVID-19 fatigue in women. Brain Sci 2022; 12: 556. doi:10.3390/brainsci12050556 35624943 PMC9139370

[28] Joli J, Buck P, Zipfel S, et al. Post-COVID-19 fatigue: a systematic review. Front Psychiatry 2022; 13: 947973. doi:10.3389/fpsyt.2022.947973 36032234 PMC9403611

[29] Wacker ME, Jörres RA, Karch A, et al. Assessing health-related quality of life in COPD: comparing generic and disease-specific instruments with focus on comorbidities. BMC Pulm Med 2016; 16: 70. doi:10.1186/s12890-016-0238-9 27160582 PMC4862227

[30] Afshari S, Ameri H, Daroudi RA, et al. Health related quality of life in adults with asthma: a systematic review to identify the values of EQ-5D-5L instrument. J Asthma 2022; 59: 1203–1212. doi:10.1080/02770903.2021.1917607 33863264

[31] Spinou A, Fragkos KC, Lee KK, et al. The validity of health-related quality of life questionnaires in bronchiectasis: a systematic review and meta-analysis. Thorax 2016; 71: 683–694. doi:10.1136/thoraxjnl-2015-207315 26869589

[32] Herrmann J, Müller K, Notz Q, et al. Prospective single-center study of health-related quality of life after COVID-19 in ICU and non-ICU patients. Sci Rep 2023; 13: 6785. doi:10.1038/s41598-023-33783-y 37100832 PMC10133285

[33] Han Q, Zheng B, Daines L, et al. Long-term sequelae of COVID-19: a systematic review and meta-analysis of one-year follow-up studies on post-COVID symptoms. Pathogens 2022; 11: 269. doi:10.3390/pathogens11020269 35215212 PMC8875269

[34] Wynberg E, van Willigen HDG, Dijkstra M, et al. Evolution of coronavirus disease 2019 (COVID-19) symptoms during the first 12 months after illness onset. Clin Infect Dis 2022; 75: e482–ee90. doi:10.1093/cid/ciab759 34473245 PMC8522402

[35] Thompson EJ, Williams DM, Walker AJ, et al. Long COVID burden and risk factors in 10 UK longitudinal studies and electronic health records. Nat Commun 2022; 13: 3528. doi:10.1038/s41467-022-30836-0 35764621 PMC9240035

[36] Daines L, Zheng B, Pfeffer P, et al. A clinical review of long-COVID with a focus on the respiratory system. Curr Opin Pulm Med 2022; 28: 174–179. doi:10.1097/MCP.0000000000000863 35131989 PMC7612723

[37] Editorial: Personalised medicine for asthma in a post-pandemic world. Lancet Respir Med 2021; 9: 1. doi:10.1016/S2213-2600(20)30582-8 33341156 PMC7831750

[38] Philip KEJ, Lonergan B, Cumella A, et al. COVID-19 related concerns of people with long-term respiratory conditions: a qualitative study. BMC Pulm Med 2020; 20: 319. doi:10.1186/s12890-020-01363-9 33298023 PMC7724437

[39] Badenoch JB, Rengasamy ER, Watson C, et al. Persistent neuropsychiatric symptoms after COVID-19: a systematic review and meta-analysis. Brain Commun 2022; 4: fcab297. doi:10.1093/braincomms/fcab297 35169700 PMC8833580

[40] Vadivel R, Shoib S, El Halabi S, et al. Mental health in the post-COVID-19 era: challenges and the way forward. Gen Psychiatr 2021; 34: e100424. doi:10.1136/gpsych-2020-100424 33644689 PMC7875255

[41] Weidinger P, Nilsson JL, Lindblad U. Medication prescribing for asthma and COPD: a register-based cross-sectional study in Swedish primary care. BMC Fam Pract 2014; 15: 54. doi:10.1186/1471-2296-15-54 24666507 PMC3987171

[42] Lucas AE, Smeenk FW, Smeele IJ, et al. Overtreatment with inhaled corticosteroids and diagnostic problems in primary care patients, an exploratory study. Fam Pract 2008; 25: 86–91. doi:10.1093/fampra/cmn006 18304973

