# Supplementary material for: Long-term impact of COVID-19 hospitalisation among individuals with pre-existing airway diseases in the UK: a multicentre, longitudinal cohort study – PHOSP-COVID
Source: ERJ Open Res. 2024 Jul 15;10(4):00982-2023. doi: 10.1183/23120541.00982-2023 (PMC11247371; doi:10.1183/23120541.00982-2023)
Supplement: Supplementary file 1 [file 00982-2023.SUPPLEMENT.pdf]

Long-term impact of COVID-19 hospitalisation among individuals with pre-existing airways diseases in the UK: a multicentre, longitudinal cohort study (PHOSP-COVID)

Omer Elneima, John R Hurst, Carlos Echevarria, Jennifer K Quint, Sam Walker, Salman Siddiqui, Petr Novotny, Paul Pfeffer, Jeremy S Brown, Manu Shankar-Hari, Hamish JC McAuley, Olivia C Leavy, Aarti Shikotra, Amisha Singapuri, Marco Sereno, Matthew Richardson, Ruth M Saunders, Victoria C Harris, Linzy Houchen-Wolloff, Neil J Greening, Ewen M Harrison, Annemarie B Docherty, Nazir I Lone, James D Chalmers, Ling-Pei Ho, Alex Horsley, Michael Marks, Krishna Poinasamy, Betty Raman, Rachael A Evans, Louise V Wain, Aziz Sheikh, Chris E Brightling, Liam G Heaney, Anthony De Soyza.

Contents

Supplementary Methods 2
Supplementary Tables 5
Supplementary Figures 18
PHOSP-COVID Collaborative Group 20
References 34

Supplementary Methods

Table SM1. Methods and thresholds for processing of variables and outcome measures presented in this analysis.

	Method
**Tables 1,2 & S4,5,7 Figure SF2 & SF3**	
Indices of Multiple Deprivation (IMD)	Obtained using postcode[1]. Different modalities used for the devolved nations.
Comorbidities	A pre-existing comorbidity was considered absent if not indicated by a 'yes' on the case report form.
Admission duration	Calculated using the hospital discharge date and the earliest admission date to the same or different hospital for the participant's COVID-19 episode.
WHO clinical progression scale	WHO classes are as follows: 3–4=no continuous supplemental oxygen needed; 5=continuous supplemental oxygen only; 6=continuous or bi-level positive airway pressure ventilation or high-flow nasal oxygen; and 7–9=invasive mechanical ventilation or other organ support e.g. vasopressors, dialysis or Extracorporeal membrane oxygenation (ECMO) [2].
Generalised Anxiety Disorder Questionnaire (GAD-7) (Anxiety)	The Generalised Anxiety Disorder (GAD-7) questionnaire is a patient reported outcome measure consisting of 7 questions with total scores ranging from 0 to 21. We used a GAD7 threshold score of > 8 to suggest at least mild-moderate anxiety.[3]
Patient Health Questionnaire (PHQ-9) (Depression)	The Patient Health Questionnaire (PHQ-9) is a patient reported outcome measure consisting of 9 questions with total scores ranging from 0 to 27. We used a PHQ-9 threshold score of ≥10 to suggest at least moderate depression.[4]
Post-Traumatic Stress Disorder Checklist for DSM V (PCL-5) Questionnaire	The Post-Traumatic Stress Disorder Checklist for DSM V (PCL-5) questionnaire is a patient reported outcome measure consisting of 20 questions assessing evidence of post-traumatic stress disorder according to the DSM V criteria. Total scores range from 0-80. We used a PCL-5 threshold score of ≥38 suggestive of a provisional diagnosis of post-traumatic stress disorder.[5,6]
Dyspnoea-12	The Dyspnoea-12 questionnaire is a patient reported outcome measure consisting of 12 questions assessing breathlessness severity incorporating both "physical" and "affective" aspects[7]. Scores range from 0 to 36 with higher scores corresponding to greater severity of breathlessness.
FACIT fatigue subscale score (FACIT)	The Functional Assessment of Chronic Illness Therapy – Fatigue (FACIT-Fatigue) scale is a patient reported outcome measure consisting of 13 questions to assess self-reported fatigue and its impact on daily activities and function.[8] Total scores range from 0-52, with lower scores corresponding to an increased burden of fatigue.[9]
Short Physical Performance Battery (SPPB)	The Short Physical Performance Battery (SPPB) test is a researcher administered assessment of physical performance and frailty. It comprises 3 components; balance, gait speed and sit to stand tests. Tests were completed according to recommended standards and training was provided to site staff by the central study team via a recorded demonstration video. SPPB total scores range from 0-12. We have reported a total SPPB score of ≤10 suggestive of underlying frailty and impaired mobility.[10-12]
Incremental Shuttle Walk Test (ISWT)	The Incremental Shuttle Walk Test (ISWT) is a researcher administered assessment of maximal physical performance and was performed according to standardised instructions with two attempts performed by participants on the same day with a 20 minutes rest between them.[13] Training was provided to site staff by the central study team via a recorded demonstration video. The best effort was reported in metres and the percent predicted value was calculated using the following reference formula accounting for gender, age and

	BMI.[14] (ISWT predicted = 1449·701 − (11·735 × age) + (241·897 × gender) − (5·686 × BMI), where male gender = 1 and female gender = 0)
Rockwood Clinical Frailty Scale (CFS)	The Rockwood Clinical Frailty Scale (CFS) is a researcher assessed scale of clinical frailty with scores ranging from 1-9 where lower scores correspond to increased frailty. We have reported CFS scores of <5 suggestive of frailty.[15]
Montreal Cognitive Assessment (MoCA)	The Montreal Cognitive Assessment (MoCA) is a researcher administered cognitive function questionnaire across 8 domains. Training was provided to site staff using standardised resources supplied online by MoCA TEST Inc.[16] The assessment was conducted in English with researchers applying their discretion to exclude participants whose command of English was insufficient to complete the test accurately. Total scores range from 0 to 30. We report total MoCA scores of <23 suggestive of at least Mild Cognitive Impairment.[17]
Spirometry and Pulmonary Function Testing	Due to COVID-19 related restrictions on aerosol-generating procedures during the study period, access to spirometry and lung function was limited. Spirometry and Pulmonary function testing were completed as per ERS/ATS recommendations.[18] Spirometry and Transfer factor values were converted to SI units if not reported as such by sites. Transfer Capacity of the Lung for the uptake of carbon monoxide (TLCO) and carbon monoxide transfer coefficient (KCO) were obtained from the best of two repeat readings. ERS Reference values were used to calculate % predicted values.[19-21] FEV_1/FVC <0·7 was used to define airflow obstruction.[22] % predicted TLCO <80% was considered indicative of impaired gas transfer.
BNP / NT-pro BNP	Brain Natriuretic Peptide (BNP) or N-terminal pro B-type Natriuretic Peptide (NT-pro BNP) were collected according to each site's routine clinically available assay as a biomarker of heart failure. Three sites submitted BNP results with all of the remaining sites submitting NT-pro BNP results. The threshold values used for BNP was ≥ 100 ng/litre [23] and for NT-pro BNP ≥ 400ng/litre [24] as suggestive of heart failure.
Glycated haemoglobin (HbA1c)	Glycated haemoglobin (HbA1c) was collected as a biomarker of current glycaemic control. We have reported HbA1c levels ≥ 6·5% as suggestive of a diagnosis of diabetes.[25]
C-Reactive Protein (CRP)	C-Reactive Protein (CRP) levels were collected as a biomarker of current systemic inflammation. Values reported as below the lower or upper limit reportable range for the assay used at the site have been included at the stated less than or more than cut off value for calculation of mean (SD) results. We have reported CRP levels > 5 mg/L as suggestive of systemic inflammation.
**Figure 2, Table S2**	
Patient-perceived recovery cluster membership	Four phenotypes of patient-perceived recovery were identified using patient symptom questionnaire, physical performance, and cognitive assessment data from the five-month visit (Dyspnoea-12, FACIT, GAD-7, PHQ-9, PCL-5, SPPB, and MoCA as continuous variables). This was completed using clustering large applications k-medoids approach where the continuous variables scores were centred, normalised, and transformed so that higher burden of disease represented higher values. [26] A Euclidean distance metric was used, and the optimal number of clusters was chosen using a silhouette plot. The four clusters include: very severe mental and physical impairment, severe mental and physical impairment, moderate mental and physical impairment with cognitive impairment, and mild.
**Table 3, S3,6,8,9**<br>**Figure 3, 4**	
Symptoms at five-month and one-year visits	Symptom severity was rated using a 0-10 visual analogue scale for Breathlessness, Cough, Fatigue, Sleep quality and Pain before COVID-19 illness and worst in the last 24 hours.
EQ-5D-5L Utility Index	The EQ-5D-5L is a five-dimension patient reported outcome questionnaire recording a patient's self-rated health state for mobility, self-care, usual activities, pain/discomfort and anxiety/depression. These scores are then mapped to a United Kingdom specific Utility Index anchored at 1 for "perfect health" and 0 for "dead" calculated from reported EQ5D-5L scores

	across the five dimensions.[27]
EQ-5D-5L VAS	The EQ-5D-5L Visual Analogue Scale is a patient reported outcome questionnaire recording the patient's self-rated health and was completed for "before your COVID-19 illness" and "your own health state today." Scores are presented as mean and standard deviation.[28]
Washington Group Short Set of Functioning Severity Continuum	The Washington Group Short Set of Functioning (WG-SS) is a patient reported outcome questionnaire using six questions to assess disability and function. Participant responses were transformed to the "Severity Continuum" by assigning scores of zero to responses "no difficulty", one to responses "some difficulty", six to responses "a lot of difficulty" and 36 to responses "cannot do at all". [29]

Supplementary Tables

Table S1: Common prescribed medications on discharge with recorded additional prescription at both research visits

	On discharge		Changes at 5-month visit		Changes at 1-year visit	
	Non-airways (n=2,082)	Airways (n=615)	Non-airways (n=1,975)	Airways (n=595)	Non-airways (n=1,621)	Airways (n=479)
**Inhalers**						
SABA	64 (3.1%)	378 (61.5%)	19 (1.0%)	60 (10.1%)	24 (1.5%)	20 (4.2%)
LAMA	8 (0.4%)	56 (9.1%)	< 5	12 (2.0%)	< 5	9 (1.9%)
LABA/LAMA	< 5	16 (2.6%)	< 5	< 5	< 5	1 (0.2%)
ICS	22 (1.1%)	89 (14.5%)	6 (0.3%)	9 (1.5%)	10 (0.6%)	6 (1.3%)
ICS/LABA	18 (0.9%)	273 (44.4%)	9 (0.5%)	62 (10.4%)	6 (0.3%)	25 (5.2%)
ICS/LABA/LAMA	0	31 (5.0%)	< 5	7 (1.2%)	< 5	3 (0.6%)
**Diuretics**	141 (6.8%)	73 (11.9%)	37 (1.9%)	15 (2.5%)	22 (1.4%)	10 (2.1%)
**Carbocisteine**	31 (1.5%)	42 (6.8%)	5 (0.3%)	11 (1.8%)	< 5	3 (0.6%)
**Montelukast**	< 5	61 (9.9%)	< 5	13 (2.2%)	< 5	7 (1.5%)
**Antidepressants**	351 (16.9%)	167 (27.2%)	86 (4.4%)	44 (7.4%)	63 (3.9%)	33 (6.9%)
**Benzodiazepines**	22 (1.1%)	9 (1.5%)	6 (0.3%)	4 (0.7%)	8 (0.5%)	0

Data are n (%). Percentages are calculated by category after exclusion of missing data for that variable. Changes at both visits refer to new prescription of class medications. SABA= Short-Acting Beta Agonist. LAMA= Long-Acting Muscarinic Antagonist. LABA= Long-Acting Beta Agonist. ICS=Inhaled Corticosteroids.

Table S2: Recovery cluster assignment at five-month stratified by presence or absence of airways disease

	n	Pre-existing airways disease (n= 595)	n	No history of airways diseases (n=1,975)	P value
Cluster assignment at five-months	554		1,851		0.000
Mild		117 (21.1%)		606 (32.7%)	
Moderate/cognitive		108 (19.5%)		435 (23.5%)	
Severe		149 (26.9%)		487 (26.3%)	
Very severe		180 (32.5%)		323 (17.5%)	

Table S3: Participants clinical characteristics at five-month stratified by the different classes of airways diseases

	n	COPD (N=136)	n	Asthma (N=428)	n	Bronchiectasis (N=31)	n	No history of airways diseases (n=1,975)	P value
Age†	136	67.2 (8.1)	428	55.7 (13.0)	31	62.1 (12.0)	1,975	57.8 (12.5)	0.000
Sex at birth	136		428		31		1,974		0.000
Male		88 (64.7%)		196 (45.8%)		22 (71.0%)		1,268 (64.2%)	
Female		48 (35.3%)		232 (54.2%)		9 (29.0%)		706 (35.8%)	
Ethnicity	136		422		31		1,965		0.000
White		130 (95.6%)		329 (78.0%)		27 (87.1%)		1,428 (72.7%)	
Non white		6 (4.4%)		93 (22.0%)		<5 (12.9%)		537 (27.3%)	
Smoking	125		389		25		1,774		0.000
Current smoker		9 (7.2%)		12 (3.1%)		<5 (8.0%)		55 (3.1%)	
Ex-smoker		100 (80.0%)		125 (32.1%)		9 (36.0%)		641 (36.1%)	
Non smoker		16 (12.8%)		252 (64.8%)		14 (56.0%)		1,078 (60.8%)	
Index of multiple deprivation index (IMD)	135		426		31		1,958		0.005
1 - most deprived		46 (34.1%)		107 (25.1%)		<5 (9.7%)		432 (22.1%)	
2		31 (23.0%)		110 (25.8%)		<5 (12.9%)		456 (23.3%)	
3		18 (13.3%)		64 (15.0%)		9 (29.0%)		351 (17.9%)	
4		17 (12.6%)		59 (13.9%)		6 (19.4%)		362 (18.5%)	
5 - least deprived		23 (17.0%)		86 (20.2%)		9 (29.0%)		357 (18.2%)	
BMI	91		303		25		1,379		
Median††		30.0 [25.9-33.2]		33.2 [28.5-38.1]		30.9 [29.2-35.0]		30.7 [27.5-35.2]	0.000
<30 kg/m2		44 (48.4%)		99 (32.7%)		9 (36.0%)		621 (45.0%)	0.001
≥30 kg/m2		47 (51.6%)		204 (67.3%)		16 (64.0%)		758 (55.0%)	
WHO clinical progression scale	136		428		31		1,975		0.032
WHO class 3-4		27 (19.9%)		67 (15.6%)		<5 (9.7%)		326 (16.5%)	
WHO class 5		66 (48.5%)		186 (43.5%)		18 (58.1%)		820 (41.5%)	
WHO class 5		34 (25.0%)		104 (24.3%)		5 (16.1%)		452 (22.9%)	
WHO class 7-9		9 (6.6%)		71 (16.6%)		5 (16.1%)		377 (19.1%)	
Median number of comorbidities††	136	3 [2-5]	428	2 [0-3]	31	2 [1-4]	1,975	1 [0-3]	0.000
Cardiovascular	136	96 (70.6%)	428	173 (40.4%)	31	18 (58.1%)	1,975	896 (45.4%)	0.000
Neuro-psychiatric	136	40 (29.4%)	428	129 (30.1%)	31	9 (29.0%)	1,975	357 (18.1%)	0.000
Admission duration, days	136	11.3 (11.5)	428	13.8 (16.7)	31	20.1 (27.3)	1,975	14.3 (18.5)	0.071
Systemic steroids	132	84 (63.6%)	404	259 (64.1%)	28	19 (67.9%)	1,880	1,037 (55.2%)	0.002
Antibiotic therapy	136	107 (78.7%)	417	333 (79.9%)	29	27 (93.1%)	1,925	1,504 (78.1%)	0.233
Anti-coagulants	129	50 (38.8%)	404	175 (43.3%)	27	14 (51.9%)	1,886	878 (46.6%)	0.222
**PROMS**									
GAD-7 total score†	125	6.1 (6.2)	398	6.8 (6.0)	29	7.7 (6.7)	1,856	4.9 (5.5)	0.001

Anxiety (GAD-7 >8)	125	39 (31.2%)	398	139 (34.9%)	29	12 (41.4%)	1,856	424 (22.8%)	0.000
PHQ-9 total score†	124	8.3 (7.2)	397	9.1 (6.8)	29	9.6 (7.8)	1,856	6.5 (6.3)	0.000
Depression (PHQ-9 ≥10)	124	45 (36.3%)	397	185 (46.6%)	29	13 (44.8%)	1,856	491 (26.5%)	0.000
Dyspnoea-12†	122	12.4 (9.8)	389	9.5 (9.6)	29	10.7 (9.4)	1,821	5.3 (7.4)	0.000
FACIT fatigue subscale score†	126	30.7 (12.6)	381	29.3 (13.9)	28	30.1 (14.5)	1,791	36.1 (12.6)	0.000
**Physical performance**									
SPPB total score†	123	8.2 (2.8)	388	9.5 (2.6)	28	9.5 (2.4)	1,803	9.9 (2.2)	0.000
SPPB ≤10 (impaired mobility)	123	94 (71.3%)	388	210 (54.1%)	28	17 (60.7%)	1,803	875 (48.5%)	0.000
ISWT distance (m)†	82	267 (156)	321	389 (265)	19	370 (189)	1,467	441 (267)	0.000
ISWT % predicted†	58	40.6 (21.8)	232	55.2 (31.6)	15	55.2 (27.9)	1,032	58.7 (29.9)	0.000
**Frailty and cognition**									
RCF ≥5	121	22 (18.2%)	393	41 (10.4%)	27	<5 (7.4%)	1,743	70 (4.0%)	0.000
SARC-F total score†	126	3.2 (2.5)	386	2.7 (2.5)	29	2.8 (2.3)	1,785	1.7 (2.1)	0.000
Corrected MoCA total score†	107	25.1 (4.1)	349	25.9 (3.7)	26	26.0 (2.6)	1,616	26.1 (3.4)	0.076
Corrected MoCA <23	107	22 (20.6%)	349	47 (13.5%)	26	5 (19.2%)	1,616	205 (12.7%)	0.101
**Lung Physiology**									
FEV1 (L) †	72	2.03 (0.67)	272	2.56 (0.75)	22	2.75 (0.74)	1,150	2.85 (0.79)	0.000
FEV1 % predicted†	68	76.0 (22.6)	252	86.5 (18.1)	21	88.2 (17.8)	1,098	91.7 (17.8)	0.000
FEV1 % predicted <80%	68	38 (55.9%)	252	90 (35.7%)	21	7 (33.3%)	1,098	255 (23.2%)	0.000
FVC (L) †	72	3.20 (0.82)	272	3.23 (0.96)	22	3.42 (1.02)	1,151	3.55 (1.03)	0.001
FVC % predicted†	68	91.4 (18.2)	252	86.3 (17.0)	21	84.4 (16.1)	1,099	89.8 (19.0)	0.025
FVC % predicted <80%	68	19 (27.9%)	252	90 (35.7%)	21	7 (33.3%)	1,099	311 (28.3%)	0.131
FEV1/FVC†	72	0.63 (0.14)	272	0.80 (0.10)	22	0.82 (0.13)	1,147	0.81 (0.09)	0.000
FEV1/FVC <0.7	72	44 (61.1%)	272	33 (12.1%)	22	<5 (13.6%)	1,147	83 (7.2%)	0.000
TLCO†	20	7.4 (3.1)	96	7.0 (1.9)	6	6.8 (1.9)	389	7.5 (2.4)	0.254
TLCO % predicted†	20	99.8 (46.2)	95	89.3 (20.5)	6	81.5 (27.5)	378	91.9 (32.5)	0.468
TLCO % predicted <80%	20	6 (30.0%)	95	34 (35.8%)	6	5 (83.3%)	378	130 (34.4%)	0.091
KCO†	20	1.3 (0.4)	100	1.5 (0.2)	7	1.4 (0.2)	393	1.4 (0.3)	0.002
KCO % predicted†	20	91.0 (28.5)	100	104.8 (14.4)	7	97.6 (15.9)	380	100.5 (20.9)	0.031
KCO % predicted <80%	20	5 (25.0%)	100	<5 (3.0%)	7	<5 (14.3%)	380	36 (9.5%)	0.011
**Biochemical Tests**									
Haemoglobin†	111	138.9 (15.4)	363	139.5 (15.6)	28	139.9 (15.9)	1,642	141.4 (15.8)	0.096
Neutrophils†	111	4.6 (1.8)	360	4.4 (1.8)	28	4.4 (1.1)	1,634	4.0 (1.5)	0.000
Eosinophils†	110	0.27 (0.26)	357	0.21 (0.20)	28	0.26 (0.22)	1,626	0.18 (0.17)	0.000
BNP/Pro-NT-BNP above threshold	82	9 (10.9%)	276	14 (5.1%)	20	<5 (10.0%)	1,197	76 (6.4%)	0.252
HbA1C ≥6.0	94	43 (45.7%)	281	98 (34.9%)	19	9 (47.4%)	1,236	427 (34.6%)	0.111
eGFR <60 (ml/min/1.73 m^2)	110	17 (15.5%)	353	37 (10.5%)	25	<5 (12.0%)	1,581	155 (9.8%)	0.297
**Systemic inflammation**									
CRP (mg/L)†	105	6.9 (8.8)	342	6.2 (9.9)	25	4.0 (3.4)	1,582	5.2 (11.2)	0.196

CRP >5 mg/L	105	39 (37.1%)	342	102 (29.8%)	25	5 (20.0%)	1,582	348 (22.0%)	0.000
CRP ≥10 mg/L	105	20 (19.0%)	342	51 (14.9%)	25	<5 (8.0%)	1,582	155 (9.8%)	0.002

Data are n (%) unless † mean (SD) or †† median [IQR]. Percentages are calculated by category after exclusion of missing data for that variable. IMD=Index of Multiple Deprivation. BMI=body-mass index. WHO classes are as follows: 3–4=no continuous supplemental oxygen needed; 5=continuous supplemental oxygen only; 6=continuous or bi-level positive airway pressure ventilation or high-flow nasal oxygen; and 7–9=invasive mechanical ventilation or other organ support. SARS-CoV-2 PCR=Severe Acute Respiratory Syndrome Coronavirus 2. Polymerase Chain Reaction. GAD7=Generalized Anxiety Disorder 7-item scale. PHQ-9=Patient Health Questionnaire-9. PCL-5=Post Traumatic Stress Disorder Checklist. FACIT fatigue=Functional Assessment of Chronic Illness Therapy Fatigue Scale. SPPB=short physical performance battery. ISWT=incremental shuttle walk test. CFS=Clinical Frailty Scale. MoCA=Montreal Cognitive Assessment. FEV1=Forced expiratory volume measured in 1 second. FVC=forced vital capacity. TLCO=transfer capacity of the lung for carbon monoxide. KCO=carbon monoxide transfer coefficient. BNP=brain natriuretic peptide. NT-BNP=N-terminal BNP. HbA1C=glycated haemoglobin. eGFR=estimated glomerular filtration rate CRP=C-reactive protein. Threshold of BNP ≥100 ng/L or NT-BNP ≥400 ng/L. Corrected MoCA adjusted for level of education. See Table SM1 for further descriptions of variables.

Table S4: Recovery, health related quality of life and symptoms burden at five-month stratified by the different classes of airways diseases

	n	COPD (N=136)	n	Asthma (N=428)	n	Bronchiectasis (N=31)	n	No history of airways diseases (n=1,975)	P value
Fully recovered from COVID-19?	118		364		27		1,693		0.007
Yes		24 (20.3%)		72 (19.8%)		4 (14.8%)		467 (27.6%)	
No		62 (52.6%)		223 (61.2%)		18 (66.7%)		912 (53.9%)	
Not sure		32 (27.1%)		69 (19.0%)		5 (18.5%)		314 (18.5%)	
Cluster assignment at 5-month	129		396		29		1,851		0.000
Mild		17 (13.2%)		93 (23.5%)		7 (24.1%)		606 (32.7%)	
Moderate/cognitive		43 (33.3%)		61 (15.4%)		4 (13.8%)		435 (23.5%)	
Severe		36 (27.9%)		106 (26.8%)		7 (24.1%)		487 (26.3%)	
Very severe		33 (25.6%)		136 (34.3%)		11 (38.0%)		323 (17.5%)	
EQ-5D-5L utility index pre-COVID†	116	0.66 (0.26)	363	0.77 (0.27)	24	0.67 (0.32)	1,667	0.84 (0.21)	0.000
EQ-5D-5L utility index at 5-month†	107	0.58 (0.27)	354	0.64 (0.29)	26	0.62 (0.29)	1,626	0.73 (0.24)	0.000
EQ-5D-5L utility index delta change†	88	-0.08 (0.23)	295	-0.14 (0.26)	21	-0.09 (0.24)	1,353	-0.11 (0.21)	0.090
EQ-5D-5L VAS pre-COVID†	118	66.8 (18.2)	352	76.3 (17.9)	21	69.3 (19.9)	1,604	81.3 (16.8)	0.000
EQ-5D-5L VAS at 5-month†	110	61.0 (20.9)	352	64.4 (21.3)	26	59.2 (22.0)	1,618	72.2 (19.2)	0.000
EQ-5D-5L VAS delta change†	93	-6.6 (20.4)	285	-12.0 (21.7)	19	-8.2 (16.3)	1,300	-9.7 (18.8)	0.090
PSQ Breathlessness pre-COVID†	106	3.9 (2.5)	362	2.2 (2.5)	25	2.7 (2.8)	1,669	0.8 (1.7)	0.000
PSQ Breathlessness at 5-month†	112	5.3 (2.6)	357	4.8 (2.9)	27	4.8 (3.0)	1,697	3.7 (2.9)	0.000
PSQ Breathlessness delta change†	105	1.4 (2.9)	352	2.6 (3.2)	24	2.0 (2.3)	1,628	2.9 (3.0)	0.000
PSQ Cough pre-COVID†	104	2.0 (2.5)	360	1.5 (2.4)	25	2.0 (2.6)	1,664	0.6 (1.5)	0.000
PSQ Cough at 5-month†	110	3.2 (2.9)	354	2.8 (2.9)	27	3.6 (3.1)	1,693	1.9 (2.6)	0.000
PSQ Cough delta change†	104	1.1 (2.7)	348	1.3 (3.2)	24	1.3 (2.8)	1,622	1.3 (2.7)	0.849
PSQ Fatigue pre-COVID†	104	3.0 (2.6)	359	1.9 (2.5)	25	2.7 (3.1)	1,664	1.4 (2.2)	0.000

PSQ Fatigue at 5-month†	109	5.5 (2.9)	354	5.7 (2.9)	27	5.8 (2.9)	1,693	4.5 (3.0)	0.000
PSQ Fatigue delta change†	103	2.5 (3.3)	347	3.8 (3.3)	24	2.9 (3.2)	1,621	3.1 (3.2)	0.001
PSQ Sleep disturbance pre-COVID†	105	3.2 (2.6)	358	2.6 (2.8)	25	2.7 (2.7)	1,663	1.9 (2.5)	0.000
PSQ Sleep disturbance at 5-month†	110	4.6 (3.0)	354	5.0 (3.0)	27	5.1 (2.9)	1,686	3.8 (3.1)	0.000
PSQ Sleep disturbance delta change†	104	1.4 (2.8)	345	2.3 (3.1)	24	2.1 (2.4)	1,616	1.9 (3.1)	0.021
PSQ Pain pre-COVID†	103	2.6 (2.9)	360	2.2 (2.9)	26	2.3 (3.2)	1,649	1.4 (2.4)	0.000
PSQ Pain at 5-month†	108	3.7 (3.2)	356	4.1 (3.4)	28	4.2 (3.6)	1,677	3.0 (3.1)	0.000
PSQ Pain delta change†	102	1.0 (2.3)	350	2.0 (3.0)	25	1.8 (2.7)	1,601	1.6 (2.8)	0.015

Missing not included in %. Number (%) unless † mean (SD). EQ-5D-5L VAS = Euroqol five level visual analogue scale 0-100. WG-SS-SCo = Washington Group Short Set of Functioning Severity Continuum. PSQ = Patient Symptoms Questionnaires. See Table SM1 for further descriptions of variables.

Table S5: Participants clinical characteristics at 1-year stratified by the different classes of airways diseases

	n	COPD (N=112)	n	Asthma (N=346)	n	Bronchiectasis (N=21)	n	No history of airways diseases (n=1,621)	p value
Age†	112	67.2 (8.1)	346	56.6 (12.3)	21	60.9 (12.8)	1621	58.9 (12.1)	0.000
Sex at birth	112		346		21		1,621		0.000
Male		71 (63.4%)		165 (47.7%)		15 (71.4%)		1,038 (64.0%)	
Female		41 (36.6%)		181 (52.3%)		6 (28.6%)		583 (36.0%)	
Ethnicity	111		341		21		1,617		0.000
White		104 (93.7%)		265 (77.7%)		19 (90.5%)		1,221 (75.5%)	
Non white		7 (6.3)		76 (22.3%)		<5 (9.5%)		396 (24.5%)	
Smoking	99		311		20		1,470		0.000
Current smoker		8 (8.1%)		9 (2.9%)		<5 (5.0%)		40 (2.7%)	
Ex-smoker		76 (76.7%)		98 (31.5%)		7 (35.0%)		549 (37.4%)	
Non smoker		15 (15.2%)		204 (65.6%)		12 (60.0%)		881 (59.9%)	
Index of multiple deprivation index (IMD)	111		346		21		1,613		0.001
1 - most deprived		38 (34.3%)		81 (23.4%)		<5 (4.8%)		346 (21.4%)	
2		24 (21.6%)		88 (25.4%)		<5 (4.8%)		347 (21.5%)	
3		17 (15.3%)		52 (15.0%)		7 (33.3%)		306 (18.9%)	
4		11 (9.9%)		49 (14.2%)		<5 (19.0%)		307 (19.1%)	
5 - least deprived		21 (18.9%)		76 (22.0%)		8 (38.1%)		307 (19.1%)	
BMI	75		252		19		1120		
Median††		31.0 [26.9-33.4]		33.0 [28.6-38.2]		30.6 [29.2-37.1]		30.9 [27.5-35.3]	0.000
<30 kg/m2		32 (42.7%)		79 (31.4%)		7 (36.8%)		495 (44.2%)	0.003
≥30 kg/m2		43 (57.3%)		173 (68.6%)		12 (63.2%)		625 (55.8%)	

WHO clinical progression scale	112		346		21		1,621		0.237
WHO class 3-4		22 (19.6%)		54 (15.6%)		2 (9.5%)		243 (15.0%)	
WHO class 5		52 (46.4%)		147 (42.5%)		11 (52.5%)		685 (42.3%)	
WHO class 5		30 (26.8%)		84 (24.3%)		4 (19.0%)		386 (23.8%)	
WHO class 7-9		8 (7.2%)		61 (17.6%)		4 (19.0%)		307 (18.9%)	
Median number of comorbidities††	112	3 [2-5]	346	2 [0-3]	21	2 [1-2]	1,621	1 [0-3]	0.000
Cardiovascular	112	80 (71.4%)	346	144 (41.6%)	21	11 (52.4%)	1,621	757 (46.7%)	0.000
Neuro-psychiatric	112	30 (26.8%)	346	99 (28.6%)	21	4 (19.1%)	1,621	298 (18.4%)	0.000
Admission duration, days†	112	11.0 (10.9)	346	14.7 (18.1)	21	18.4 (30.1)	1,621	14.8 (19.1)	0.155
Systemic steroids	111	72 (64.9%)	324	206 (63.6%)	19	12 (63.2%)	1,540	865 (56.2%)	0.035
Antibiotic therapy	112	86 (76.8%)	337	265 (78.6%)	20	18 (90%)	1,577	1,238 (78.5%)	0.622
Anti-coagulants	106	41 (38.7%)	324	143 (44.1%)	18	7 (38.9%)	1,543	737 (47.8%)	0.192
Recovered from COVID-19?	96		286		21		1384		0.000
Yes		21 (21.9%)		58 (20.3%)		3 (14.3%)		459 (33.2%)	
No		48 (50.0%)		164 (57.3%)		13 (61.9%)		638 (46.1%)	
Not sure		27 (28.1%)		64 (22.4%)		5 (23.8%)		287 (20.7%)	
**PROMS**									
GAD-7 total score†	106	5.5 (5.9)	320	6.1 (6.1)	21	5.2 (6.2)	1,503	4.8 (5.5)	0.001
Anxiety (GAD-7 >8)	106	30 (28.3%)	320	94 (29.4%)	21	6 (28.6%)	1,503	331 (22.0%)	0.023
PHQ-9 total score†	106	7.0 (6.1)	316	7.7 (6.7)	21	6.8 (7.6)	1,504	6.1 (6.3)	0.001
Depression (PHQ-9 ≥10)	106	28 (26.4%)	316	104 (32.9%)	21	6 (28.6%)	1,504	371 (24.7%)	0.026
Dyspnoea-12†	102	11.0 (8.9)	307	7.7 (8.6)	21	7.9 (8.6)	1,462	4.9 (7.2)	0.000
FACIT fatigue subscale score†	95	32.6 (11.2)	288	32.3 (13.9)	19	37.7 (10.9)	1,400	36.7 (12.4)	0.000
**Physical performance**									
SPPB total score†	87	8.8 (2.4)	292	9.5 (2.6)	18	9.6 (2.6)	1,397	10.2 (2.1)	0.000
SPPB ≤10 (impaired mobility)	87	62 (71.3%)	292	157 (53.8%)	18	9 (50.0%)	1,397	632 (45.2%)	0.000
ISWT distance (m) †	57	273 (170)	223	422 (267)	15	424 (261)	1104	456 (267)	0.000
ISWT % predicted†	41	40.7 (24.8)	172	56.9 (31.2)	13	57.6 (33.1)	796	60.8 (30.3)	0.000
**Frailty and cognition**									
RCF ≥5	98	18 (18.4%)	306	26 (8.5%)	18	1 (5.6%)	1,463	59 (4.0%)	0.000
SARC-F total score†	95	2.9 (2.4)	289	2.3 (2.5)	19	1.9 (1.9)	1,405	1.7 (2.1)	0.000
Corrected MoCA total score†	86	25.9 (3.4)	273	26.9 (3.2)	20	27.9 (2.3)	1,303	26.6 (3.3)	0.030
Corrected MoCA <23	86	14 (16.3%)	273	24 (8.8%)	20	<5 (5.0%)	1,303	139 (10.7%)	0.208
**Lung Physiology**									
FEV1 (L) †	44	2.10 (0.66)	186	2.60 (0.81)	11	2.96 (0.79)	840	2.89 (0.80)	0.000
FEV1 % predicted†	41	79.9 (23.3)	179	87.4 (20.6)	11	97.3 (16.4)	820	93.2 (17.4)	0.000
FEV1 % predicted <80%	41	18 (43.9%)	179	65 (36.3%)	11	1 (9.1%)	820	173 (21.1%)	0.000
FVC (L)†	44	3.19 (0.83)	186	3.33 (1.01)	11	3.75 (0.94)	843	3.61 (1.01)	0.001
FVC % predicted†	41	92.6 (19.6)	179	88.6 (20.9)	11	96.4 (11.3)	823	91.2 (17.8)	0.237

FVC % predicted <80%	41	13 (31.7%)	179	64 (35.8%)	11	1 (9.1%)	823	188 (22.8%)	0.001
FEV1/FVC†	44	0.66 (0.12)	186	0.79 (0.20)	11	0.79 (0.08)	840	0.81 (0.11)	0.000
FEV1/FVC <0.7	44	22 (50.0%)	186	32 (17.2%)	11	1 (9.1%)	840	63 (7.5%)	0.000
TLCO†	17	7.08 (2.88)	55	7.81 (2.21)	4	7.37 (1.11)	264	7.69 (2.36)	0.722
TLCO % predicted†	17	98.2 (45.8)	55	96.6 (21.7)	4	94.1 (9.1)	261	94.9 (29.7)	0.958
TLCO % predicted <80%	17	6 (35.3%)	55	12 (21.8%)	4	0	261	60 (23.0%)	0.445
KCO†	17	1.30 (0.33)	59	1.52 (0.25)	4	1.28 (0.19)	273	1.44 (0.27)	0.009
KCO % predicted†	17	92.4 (22.2)	59	105.3 (16.2)	4	92.0 (11.4)	270	100.0 (17.3)	0.027
KCO % predicted <80%	17	5 (29.4%)	59	4 (6.8%)	4	0	270	24 (8.9%)	0.030
**Biochemical Tests**									
Haemoglobin†	89	140.8 (15.9)	275	139.0 (15.8)	19	143.1 (13.3)	1,279	141.6 (14.8)	0.069
Neutrophils†	89	5.0 (2.2)	274	4.5 (2.2)	18	4.8 (1.3)	1,275	4.0 (1.5)	0.000
Eosinophils†	89	0.23 (0.19)	274	0.21 (0.18)	18	0.28 (0.16)	1,273	0.19 (0.18)	0.006
BNP/Pro-NT-BNP above threshold	53	8 (15.1%)	169	13 (7.7%)	15	2 (13.3%)	824	68 (8.3%)	0.310
HbA1C ≥6.0	64	33 (51.6%)	199	66 (33.2%)	16	8 (50.0%)	1,008	355 (35.2%)	0.029
eGFR <60 (ml/min/1.73 m^2)	85	12 (14.1%)	261	32 (12.3%)	13	2 (15.4%)	1,227	153 (12.5%)	0.959
**Systemic inflammation**									
CRP (mg/L) †	79	5.8 (5.8)	277	5.9 (6.6)	18	5.4 (6.7)	1,260	4.9 (7.0)	0.143
CRP >5 mg/L	79	26 (32.9%)	277	83 (29.9%)	18	5 (27.8%)	1,260	278 (22.1%)	0.009
CRP ≥10 mg/L	79	10 (12.7%)	277	42 (15.2%)	18	1 (5.6%)	1,260	120 (9.5%)	0.037

Data are n (%) unless † mean (SD) or †† median [IQR]. Percentages are calculated by category after exclusion of missing data for that variable. IMD=Index of Multiple Deprivation. BMI=body-mass index. WHO classes are as follows: 3–4=no continuous supplemental oxygen needed; 5=continuous supplemental oxygen only; 6=continuous or bi-level positive airway pressure ventilation or high-flow nasal oxygen; and 7–9=invasive mechanical ventilation or other organ support. SARS-CoV-2 PCR=Severe Acute Respiratory Syndrome Coronavirus 2. Polymerase Chain Reaction. GAD7=Generalized Anxiety Disorder 7-item scale. PHQ-9=Patient Health Questionnaire-9. PCL-5=Post Traumatic Stress Disorder Checklist. FACIT fatigue=Functional Assessment of Chronic Illness Therapy Fatigue Scale. SPPB=short physical performance battery. ISWT=incremental shuttle walk test. CFS=Clinical Frailty Scale. MoCA=Montreal Cognitive Assessment. FEV1=Forced expiratory volume measured in 1 second. FVC=forced vital capacity. TLCO=transfer capacity of the lung for carbon monoxide. KCO=carbon monoxide transfer coefficient. BNP=brain natriuretic peptide. NT-BNP=N-terminal BNP. HbA1C=glycated haemoglobin. eGFR=estimated glomerular filtration rate CRP=C-reactive protein. Threshold of BNP ≥100 ng/L or NT-BNP ≥400 ng/L. Corrected MoCA adjusted for level of education. See Table SM1 for further descriptions of variables.

Table S6: Recovery, health related quality of life and symptoms burden at the 1-year stratified by the different classes of airways diseases

	n	COPD (N=112)	n	Asthma (N=346)	n	Bronchiectasis (N=21)	n	No history of airways diseases (n=1621)	p value
Recovered from COVID-19?	96		286		21		1384		0.000
Yes		21 (21.9%)		58 (20.3%)		3 (14.3%)		459 (33.2%)	
No		48 (50.0%)		164 (57.3%)		13 (61.9%)		638 (46.1%)	
Not sure		27 (28.1%)		64 (22.4%)		5 (23.8%)		287 (20.7%)	
EQ-5D-5L utility index pre-COVID†	101	0.66 (0.25)	302	0.77 (0.27)	19	0.77 (0.27)	1399	0.84 (0.21)	0.000
EQ-5D-5L utility index at 1-year†	94	0.61 (0.24)	280	0.65 (0.28)	16	0.63 (0.29)	1350	0.73 (0.25)	0.000
EQ-5D-5L utility index delta change†	84	-0.05 (0.18)	240	-0.12 (0.25)	14	-0.02 (0.29)	1160	-0.11 (0.22)	0.031
EQ-5D-5L VAS pre-COVID†	101	67.7 (18.9)	293	77.2 (18.3)	16	71.3 (21.4)	1342	81.1 (16.6)	0.000
EQ-5D-5L VAS at 1-year†	95	63.8 (19.6)	274	68.4 (21.5)	15	67.1 (24.8)	1347	71.3 (20.4)	0.002
EQ-5D-5L VAS delta change†	84	-2.6 (17.2)	228	-11.1 (20.2)	11	0.1 (29.2)	1112	-10.2 (19.7)	0.002
PSQ Breathlessness pre-COVID†	56	3.9 (2.4)	183	2.3 (2.3)	17	1.6 (1.9)	837	0.8 (1.6)	0.000
PSQ Breathlessness at 1-year†	90	4.3 (2.6)	289	3.5 (2.7)	19	2.7 (2.9)	1372	2.4 (2.5)	0.000
PSQ Breathlessness delta change†	51	0.6 (2.6)	175	1.5 (2.8)	17	0.9 (2.4)	805	2.0 (2.7)	0.000
PSQ Cough pre-COVID†	56	2.2 (2.7)	183	1.2 (1.9)	17	1.6 (2.1)	836	0.6 (1.5)	0.000
PSQ Cough at 1-year†	90	2.5 (2.4)	286	1.9 (2.5)	19	1.9 (2.6)	1368	1.3 (2.1)	0.000
PSQ Cough delta change†	51	0.3 (2.7)	173	0.8 (2.4)	17	0.2 (1.8)	801	0.9 (2.3)	0.149
PSQ Fatigue pre-COVID†	56	2.9 (2.6)	183	1.8 (2.3)	17	2.2 (2.6)	831	1.3 (2.0)	0.000
PSQ Fatigue at 1-year†	88	4.2 (2.6)	289	4.3 (3.1)	19	3.8 (3.0)	1369	3.3 (2.9)	0.000
PSQ Fatigue delta change†	51	1.5 (3.2)	175	2.8 (3.3)	17	1.7 (3.6)	799	2.4 (3.1)	0.037
PSQ Sleep disturbance pre-COVID†	54	3.3 (3.1)	184	2.4 (2.6)	17	1.2 (1.6)	836	1.8 (2.4)	0.000
PSQ Sleep disturbance at 1-year†	89	4.0 (3.1)	290	4.0 (3.1)	19	2.5 (2.2)	1,368	3.3 (3.0)	0.000
PSQ Sleep disturbance delta change†	50	0.7 (2.6)	176	1.8 (3.0)	17	1.1 (2.3)	805	1.6 (2.9)	0.000
PSQ Pain pre-COVID†	56	3.1 (2.9)	180	2.2 (2.7)	17	1.5 (2.3)	836	1.4 (2.3)	0.000
PSQ Pain at 1-year†	90	3.2 (3.0)	287	3.4 (3.1)	19	2.5 (3.0)	1,363	2.5 (2.8)	0.000
PSQ Pain delta change†	51	0.5 (2.6)	171	1.4 (2.8)	17	0.9 (2.4)	800	1.3 (2.6)	0.145

Missing not included in %. Number (%) unless † mean (SD). EQ-5D-5L VAS = Euroqol five level visual analogue scale 0-100. WG-SS-SCo = Washington Group Short Set of Functioning Severity Continuum. PSQ = Patient Symptoms Questionnaires. See Table SM1 for further descriptions of variables.

Table S7: The clinical characteristic of the participants with pre-existing airways stratified by recovery status at both the five-month and one-year research visits.

	5-month visit					1-year visit				
	n	Recovered (n=100)	n	Not recovered (n=409)	P value	n	Recovered (n=82)	n	Not recovered (n=321)	p value
**PROMS**										
GAD-7 total score†	92	4.6 (5.8)	389	7.4 (6.2)	0.001	82	3.2 (5.2)	308	6.7 (6.1)	0.000
Anxiety (GAD-7 >8)	92	19 (20.7%)	389	150 (38.6%)	0.001	82	11 (13.4%)	308	103 (33.4%)	0.000
PHQ-9 total score†	92	5.7 (6.2)	389	9.9 (6.7)	0.000	82	3.9 (5.2)	305	8.4 (6.6)	0.009
Depression (PHQ-9 ≥10)	92	19 (20.7%)	389	196 (50.4%)	0.000	82	11 (13.4%)	305	109 (35.7%)	0.000
PCL-5 total score†	92	10.7 (14.7)	388	22.9 (19.4)	0.000	81	7.9 (12.1)	304	19.5 (18.5)	0.000
PTSD (PCL-5 ≥38)	92	7 (7.6%)	388	90 (23.2%)	0.001	81	4 (4.9%)	304	56 (18.4%)	0.003
Dyspnoea-12†	89	5.2 (7.5)	382	11.7 (9.9)	0.000	82	4.1 (7.5)	292	9.6 (8.7)	0.000
FACIT fatigue subscale score†	90	39.0 (11.8)	380	27.0 (13.0)	0.000	80	41.1 (10.5)	289	30.3 (12.7)	0.000
**Physical performance**										
SPPB total score†	93	9.8 (2.5)	370	8.0 (2.7)	0.006	63	10.1 (2.5)	278	9.2 (2.4)	0.006
SPPB ≤10 (impaired mobility)	93	47 (50.5%)	370	237 (64.1%)	0.017	63	27 (42.9%)	278	174 (62.6%)	0.004
ISWT distance (m)†	63	472 (317)	293	327 (210)	0.000	47	483 (246)	211	352 (235)	0.001
ISWT % predicted†	45	64.9 (36.0)	214	48.9.0 (27.9)	0.001	33	59.3 (26.3)	169	51.0 (30.6)	0.147
**Frailty and cognition**										
RCF ≥5	91	6 (6.6%)	381	52 (13.7%)	0.066	73	7 (9.6%)	292	30 (10.3%)	0.862
SARC-F total score†	92	2.3 (2.6)	383	3.0 (2.5)	0.009	79	1.4 (2.3)	290	2.8 (2.4)	0.000
Corrected MoCA total score†	75	25.5 (3.5)	340	25.6 (3.8)	0.811	62	26.2 (4.1)	263	26.8 (3.0)	0.186
Corrected MoCA <23	75	12 (16.0%)	340	56 (16.5%)	0.921	62	8 (12.9%)	237	26 (9.9%)	0.485
**Lung Physiology**										
FEV1 (L)†	54	2.40 (0.81)	267	2.47 (0.75)	0.557	37	2.59 (0.78)	165	2.50 (0.82)	0.549
FEV1 % predicted†	53	82.3 (20.0)	247	84.9 (19.2)	0.387	35	86.9 (15.6)	161	85.8 (22.8)	0.805
FEV1 % predicted <80%	53	24 (45.3%)	247	97 (39.3%)	0.418	35	11 (31.4%)	161	61 (37.9%)	0.472
FVC (L)†	54	3.18 (0.92)	267	3.22 (0.92)	0.764	37	3.49 (0.93)	165	3.28 (0.99)	0.253
FVC % predicted†	53	86.6 (18.4)	247	87.0 (17.0)	0.874	35	93.5 (15.4)	161	88.1 (21.4)	0.166
FVC % predicted <80%	53	14 (26.4%)	247	87 (35.2%)	0.218	35	8 (22.9%)	161	61 (37.9%)	0.092
FEV1/FVC†	54	0.75 (0.13)	267	0.77 (0.12)	0.383	37	0.74 (0.10)	165	0.76 (0.13)	0.394
FEV1/FVC <0.7	54	14 (25.9%)	267	55 (20.6%)	0.385	37	9 (24.3%)	165	38 (23.0%)	0.881
TLCO†	13	8.18 (2.09)	105	6.93 (2.12)	0.048	12	8.98 (2.45)	54	7.17 (2.19)	0.014
TLCO % predicted†	13	100.6 (27.9)	105	89.8 (26.8)	0.178	12	109.2 (32.7)	54	92.3 (28.1)	0.074
TLCO % predicted <80%	13	3 (23.1%)	105	40 (38.1%)	0.289	12	1 (8.3%)	54	17 (31.5%)	0.103
KCO†	13	1.60 (0.28)	110	1.48 (0.28)	0.152	12	1.52 (0.31)	58	1.43 (0.28)	0.297
KCO % predicted†	13	107.5 (15.4)	110	102.1 (18.1)	0.309	12	102.9 (18.7)	58	99.7 (17.7)	0.575

KCO % predicted <80%	13	1 (7.7%)	110	7 (6.7%)	0.854	12	1 (8.3%)	58	8 (13.8%)	0.607
**Biochemical Tests**										
Haemoglobin†	82	139.5 (17.3)	352	139.4 (14.4)	0.955	57	139.8 (17.9)	269	138.4 (18.4)	0.596
Neutrophils†	82	4.22 (1.63)	351	4.53 (1.85)	0.158	57	4.91 (3.11)	267	4.57 (2.00)	0.301
Eosinophils†	82	0.27 (0.27)	348	0.21 (0.17)	0.011	56	0.28 (0.23)	267	0.21 (0.17)	0.016
BNP/Pro-NT-BNP above threshold	65	7 (10.8%)	263	17 (6.5%)	0.233	35	6 (17.1%)	169	15 (8.9%)	0.143
HbA1C ≥6.0	64	23 (35.9%)	277	113 (40.8%)	0.475	36	12 (33.3%)	202	81 (40.1%)	0.443
eGFR <60 (ml/min/1.73 m^2)	80	13 (16.3%)	344	40 (11.6%)	0.260	54	9 (16.7%)	249	31 (12.5%)	0.407
**Systemic inflammation**										
CRP (mg/L)†	80	7.1 (15.8)	331	5.8 (7.3)	0.362	58	4.8 (4.8)	259	5.6 (6.2)	0.362
CRP >5 mg/L	80	18 (22.5%)	331	101 (30.5%)	0.156	58	13 (22.4%)	259	77 (29.7%)	0.264
CRP ≥10 mg/L	80	11 (13.8%)	331	49 (14.8%)	0.811	58	7 (12.1%)	259	32 (12.4%)	0.952
**HRQoL, symptoms**										
EQ-5D-5L utility index pre-COVID†	85	0.80 (0.25)	347	0.71 (0.28)	0.005	69	0.83 (0.18)	286	0.72 (0.28)	0.002
EQ-5D-5L utility index at the visit†	82	0.76 (0.25)	344	0.58 (0.28)	0.000	76	0.79 (0.26)	284	0.60 (0.27)	0.000
EQ-5D-5L utility index delta change†	68	- 0.04 (0.23)	285	- 0.14 (0.26)	0.004	63	- 0.02 (0.17)	250	- 0.11 (0.25)	0.007
EQ-5D-5L VAS pre-COVID†	85	79.5 (18.0)	338	71.6 (19.1)	0.001	68	80.0 (17.5)	273	73.6 (19.2)	0.013
EQ-5D-5L VAS at the visit†	83	74.5 (18.4)	347	59.9 (21.3)	0.000	74	77.4 (17.3)	280	63.9 (21.3)	0.000
EQ-5D-5L VAS delta change†	69	- 4.9 (14.9)	281	- 12.6 (23.0)	0.009	62	- 2.2 (16.4)	238	- 10.4 (21.4)	0.006
WG-SS disability	96	18 (18.8%)	402	154 (38.3%)	0.000	81	9 (11.1%)	318	119 (37.4%)	0.000
PSQ Breathlessness pre-COVID†	94	2.3 (2.5)	380	2.7 (2.6)	0.188	46	2.2 (2.1)	192	2.7 (2.5)	0.218
PSQ Breathlessness at the visit†	95	3.0 (2.9)	387	5.5 (2.6)	0.000	78	1.9 (2.1)	312	4.1 (2.7)	0.000
PSQ Breathlessness delta change†	93	0.7 (2.8)	374	2.8 (3.0)	0.000	46	0.0 (2.0)	191	1.6 (2.8)	0.001
PSQ Cough pre-COVID†	92	1.4 (2.2)	378	1.7 (2.4)	0.240	46	1.0 (1.9)	193	1.5 (2.2)	0.144
PSQ Cough at the visit†	95	1.8 (2.5)	382	3.2 (3.0)	0.000	78	0.9 (1.7)	309	2.3 (2.6)	0.000
PSQ Cough delta change†	92	0.5 (2.7)	370	1.5 (3.1)	0.003	46	-0.2 (1.8)	190	0.8 (2.4)	0.011
PSQ Fatigue pre-COVID†	94	1.9 (2.5)	376	2.3 (2.7)	0.158	46	2.1 (2.6)	193	2.0 (2.4)	0.804
PSQ Cough at the visit†	94	3.2 (2.9)	382	6.3 (2.6)	0.000	78	1.9 (2.2)	310	4.9 (2.8)	0.000
PSQ fatigue delta change†	93	1.4 (2.8)	367	4.0 (3.3)	0.000	46	0.2 (2.6)	192	3.0 (3.3)	0.000

Data are n (%) unless † mean (SD). Percentages are calculated by category after exclusion of missing data for that variable. GAD7=Generalized Anxiety Disorder 7-item scale. PHQ-9=Patient Health Questionnaire-9. PCL-5=Post Traumatic Stress Disorder Checklist. FACIT fatigue=Functional Assessment of Chronic Illness Therapy Fatigue Scale. SPPB=short physical performance battery. ISWT=incremental shuttle walk test. CFS=Clinical Frailty Scale. MoCA=Montreal Cognitive Assessment. FEV1=Forced expiratory volume measured in 1 second. FVC=forced vital capacity. TLCO=transfer capacity of the lung for carbon monoxide. KCO=carbon monoxide transfer coefficient. BNP=brain natriuretic peptide. NT-BNP=N-terminal BNP. HbA1C=glycated haemoglobin. eGFR=estimated glomerular filtration rate CRP=C-reactive protein. HRQoL=Health Related Quality of Life. EQ-5D-5L VAS = Euroqol five level visual analogue scale 0-100. WG-SS-SCo = Washington Group Short Set of Functioning Severity Continuum. PSQ = Patient Symptoms Questionnaires. Threshold of BNP ≥100 ng/L or NT-BNP ≥400 ng/L. Corrected MoCA adjusted for level of education. See Table SM1 for further descriptions of variables.

Table S8: Multivariable logistic regression predicting recovery at 1-year in the airways group.

	n	Recovered (n=82)	n	Not recovered (n=321)	p value	Univariate OR (95% CI, p)	Multivariate OR (95% CI, p)
Age†	82	61.5 (14.9)	321	59.5 (11.1)	0.181	1.01 (0.99-1.04, p= 0.181	
Age as a factor	82		321		0.014		
<50		17 (20.7%)		55 (17.1%)		2.25 (1.03-4.91, p=0.041)	1.61 (0.54-4.78, p=0.394)
50-59		14 (17.1%)		102 (31.8%)		-	-
60-69		26 (31.7%)		107 (33.3%)		1.77 (0.88-3.58, p=0.112)	1.57 (0.63-3.88, p=0.329)
+70		25 (30.5%)		57 (17.8%)		3.19 (1.54-6.63, p=0.002)	2.48 (0.87-7.09, p=0.088)
Sex at birth	82		321		0.036		
Male		54 (65.9%)		170 (52.9%)		-	-
Female		28 (34.1%)		151 (47.1%)		0.58 (0.35-0.97, p=0.037)	0.50 (0.24-1.03, p=0.059)
Ethnicity	82		317		0.356		
White		65 (79.3%)		265 (83.6%)		-	
Non-White		17 (20.7%)		52 (16.4%)		1.33 (0.72-2.46, p=0.357)	1.74 (0.77-3.96. p=0.184)
Index of multiple deprivation index (IMD)	82		320		0.823		
1 - most deprived		18 (21.9%)		87 (27.2%)		0.65 (0.30-1.43, p=0.284)	0.73 (0.24-2.29, p=0.595)
2		19 (23.2%)		75 (23.4%)		0.80 (0.36-1.74, p=0.569)	0.61 (0.19-1.97, p=0.412)
3		14 (17.1%)		44 (13.8%)		-	
4		11 (13.4%)		46 (14.4%)		0.75 (0.31-1.83, p=0.530)	0.73 (0.21-2.48, p=0.612)
5 - least deprived		20 (24.4%)		68 (21.2%)		0.92 (0.42-2.01, p=0.844)	1.07 (0.36-3.18, p=0.899)
BMI, median value††	52	29.5 [27.1-31.7]	252	32.9 [28.7-37.6]	0.010	0.94 (0.89-0.99, p=0.014)	0.97 (0.92-1.02, p=0.240)
Median number of comorbidities††	82	2 [0-3]	321	2 [1-4]	0.164	0.92 (0.82-1.03, p=0.155)	0.96 (0.73-1.27, p=0.776)
0		24 (29.3%)		66 (20.6%)	0.233	-	-
1		15 (18.3%)		62 (19.3%)		0.67 (0.32-1.38, p=0.275)	0.80 (0.29-2.20, p=0.669)
+2		43 (52.4%)		193 (60.1%)		0.61 (0.35-1.09, p=0.093)	0.82 (0.24-2.79, p=0.752)
Neuro-psychiatric	82	12 (14.6%)	321	99 (30.8%)	0.003	0.38 (0.19-0.74, p=0.004)	0.51 (0.18-1.48, p=0.217)
Admission duration, days†	82	11.4 (12.5)	321	14.6 (18.6)	0.138	0.99 (0.97-1.00, p=0.141)	0.99 (0.95-1.02, p=0.449)
WHO clinical progression scale	82		321		0.084		
WHO class 3-4		11 (13.4%)		56 (17.4%)		-	-
WHO class 5		39 (47.6%)		136 (42.4%)		1.46 (0.70-3.05, p=0.315)	1.04 (0.39-2.79, p=0.939)
WHO class 6		25 (30.5%)		71 (22.1%)		1.79 (0.81-3.95, p=0.148)	2.09 (0.69-6.37, p=0.196)
WHO class 7-9		7 (8.5%)		58 (18.1%)		0.61 (0.22-1.70, p=0.348)	0.46 (0.06-3.50, p=0.455)
Systemic steroids	80	50 (62.5%)	301	185 (61.5%)	0.865	1.05 (0.63-1.74, p=0.865)	1.23 (0.61-2.49, p=0.558)
Antibiotic therapy	81	64 (79.0%)	312	246 (78.9%)	0.974	1.01 (0.55-1.84, p=0.974)	
Anti-coagulants	79	33 (41.8%)	293	128 (43.7%)	0.761	0.92 (0.56-1.53, p=0.761)	

Data are n (%) unless † mean (SD) or †† median [IQR]. Percentages are calculated by category after exclusion of missing data for that variable. IMD=Index of Multiple Deprivation. BMI=body-mass index. WHO classes are as follows: 3–4=no continuous supplemental oxygen needed; 5=continuous supplemental oxygen only; 6=continuous or bi-level positive airway pressure ventilation or high-flow nasal oxygen; and 7–9=invasive mechanical ventilation or other organ support.

Table S9: Multivariable logistic regression predicting recovery at 1-year in the non-airways group.

	n	Recovered (n=459)	n	Not recovered (n=925)	p value	Univariate OR (95% CI, p)	Multivariate OR (95% CI, p)
Age†	459	59.4 (13.5)	925	58.8 (11.3)	0.432	1.00 (0.99-1.01, p= 0.431)	
Age as a factor	459		925		0.000		
<50		110 (24.0%)		173 (18.7%)		1.82 (1.32-2.53, p=0.000)	1.44 (0.93-2.21, p=0.101)
50-59		105 (22.9%)		301 (32.5%)		-	-
60-69		130 (28.3%)		281 (30.4%)		1.33 (0.98-1.78, p=0.069)	1.07 (0.72-1.59, p=0.731)
+70		114 (24.8%)		170 (18.4%)		1.92 (1.39-2.66, p=0.000)	1.55 (0.99-2.43, p=0.054)
Sex at birth	459		925		0.012		
Male		317 (69.1%)		575 (62.2%)		-	-
Female		142 (30.9%)		350 (37.8%)		0.74 (0.58-0.93, p=0.012)	0.72 (0.52-0.99, p=0.042)
Ethnicity	458		923		0.000		
White		312 (68.1%)		739 (80.1%)		-	-
Non-White		146 (31.9%)		184 (19.9%)		1.88 (1.46-2.42, p=0.000)	1.92 (1.35-2.72, p=0.000)
Index of multiple deprivation index (IMD)	454		923		0.272		
1 - most deprived		84 (18.5%)		220 (23.8%)		0.72 (0.50-1.03, p=0.069)	0.71 (0.46-1.11, p=0.137)
2		96 (21.2%)		189 (20.5%)		0.95 (0.67-1.36, p=0.791)	0.90 (0.57-1.43, p=0.668)
3		89 (19.6%)		167 (18.1%)		-	-
4		94 (20.7%)		178 (19.3%)		0.99 (0.69-1.42, p=0.960)	1.15 (0.73-1.81, p=0.540)
5 - least deprived		91 (20.0%)		169 (18.3%)		1.01 (0.70-1.45, p=0.955)	1.04 (0.66-1.65, p=0.856)
BMI, median††	318	29.4 [26.7-33.4]	646	31.6 [28.0-35.6]	0.000	0.95 (0.93-0.98, p=0.000)	0.97 (0.95-0.99, p=0.046)
Median number of comorbidities††	459	1 [0-3]	925	2 [0-3]	0.005	0.91 (0.85-0.97, p=0.002)	1.02 (0.88-1.18, p=0.809)
0		160 (34.9%)		265 (28.6%)	0.029	-	-
1		100 (21.8%)		194 (21.0%)		0.85 (0.63-1.17, p=0.319)	1.05 (0.69-1.62, p=0.813)
+2		199 (43.3%)		466 (50.4%)		0.71 (0.55-0.91, p=0.008)	0.79 (0.45-1.38, p=0.403)
Neuro-psychiatric	459	48 (10.5%)	925	200 (21.6%)	0.000	0.42 (0.30-0.59, p=0.000)	0.58 (0.35-0.96, p=0.034)
Admission duration, days†	459	12.0 (16.0)	925	16.3 (20.8)	0.000	0.99 (0.98-0.99, p=0.000)	0.99 (0.98-1.00, p=0.236)
WHO clinical progression scale	459		925		0.000		
WHO class 3-4		67 (14.6%)		137 (14.8%)		-	-
WHO class 5		222 (48.4%)		375 (40.5%)		1.21 (0.87-1.69, p=0.265)	1.22 (0.78-1.89, p=0.384)
WHO class 6		112 (24.4%)		205 (22.2%)		1.12 (0.77-1.62, p=0.559)	1.10 (0.66-1.84, p=0.711)

WHO class 7-9		58 (12.6%)		208 (22.5%)		0.57 (0.38-0.86, p=0.008)	0.65 (0.33-1.26, p=0.198)
Systemic steroids	439	238 (54.2%)	872	483 (55.4%)	0.686	0.95 (0.76-1.20, p=0.686)	1.02 (0.74-1.40, p=0.904)
Antibiotic therapy	445	341 (76.6%)	902	718 (79.6%)	0.211	0.84 (0.64-1.10, p=0.211)	
Anti-coagulants	440	210 (47.7%)	875	425 (48.6%)	0.773	0.97 (0.77-1.22, p=0.773)	

Data are n (%) unless † mean (SD) or †† median [IQR]. Percentages are calculated by category after exclusion of missing data for that variable. IMD=Index of Multiple Deprivation. BMI=body-mass index. WHO classes are as follows: 3–4=no continuous supplemental oxygen needed; 5=continuous supplemental oxygen only; 6=continuous or bi-level positive airway pressure ventilation or high-flow nasal oxygen; and 7–9=invasive mechanical ventilation or other organ support

Supplementary Figures

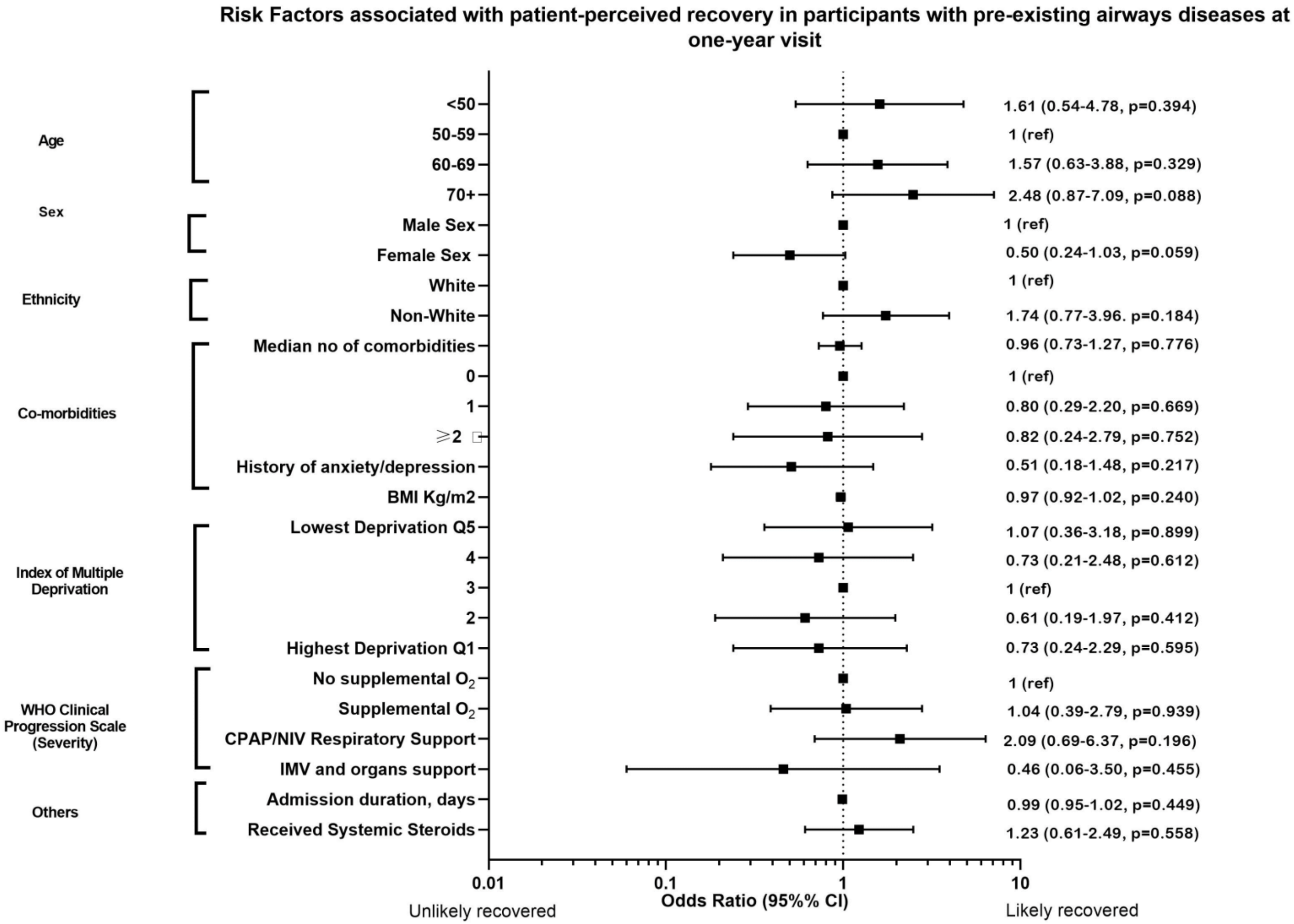

Figure SF1. Risk factors associated with patient perceived recovery in the airways group at one year visit. BMI=Body mass index. CPAP= Continuous positive airway pressure. NIV= Non-invasive ventilation. IMV= Invasive mechanical ventilation.

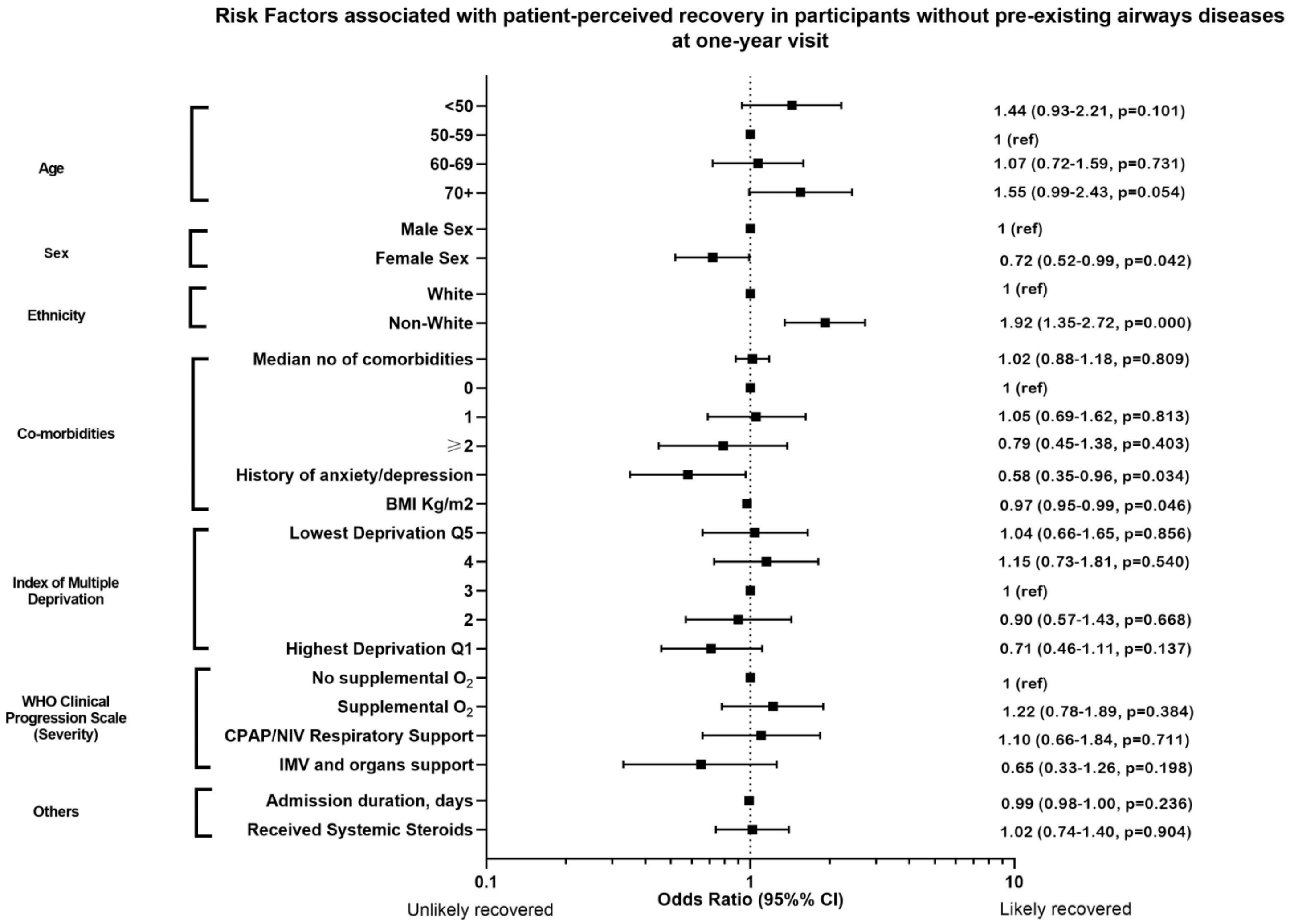

Figure SF2. Risk factors associated with patient perceived recovery in the non-airways group at one-year visit. BMI=Body mass index. CPAP= Continuous positive airway pressure. NIV= Non-invasive ventilation. IMV= Invasive mechanical ventilation.

PHOSP-COVID Collaborative Group

Core Management Group

Chief Investigator C E Brightling, *Members* R A Evans (Lead Co-I), L V Wain (Lead Co-I), J D Chalmers, V C Harris, L P Ho, A Horsley, M Marks, K Poinasamy, B Raman, A Shikotra, A Singapuri

PHOSP-COVID Study Central Coordinating Team

C E Brightling (Chief Investigator), R A Evans (*Lead Co-I*), L V Wain (*Lead Co-I*), R Dowling, C Edwardson, O Elneima, S Finney, N J Greening, B Hargadon, V C Harris, L Houchen--Wolloff, O C Leavy, H J C McAuley, C Overton, T Plekhanova, R M Saunders, M Sereno, A Singapuri, A Shikotra, C Taylor, S Terry, C Tong, B Zhao

Steering Committee

Co-chairs D Lomas, E Sapey, *Institution representatives* C Berry, C E Bolton, N Brunskill, E R Chilvers, R Djukanovic, Y Ellis, D Forton, N French, J George, N A Hanley, N Hart, L McGarvey, N Maskell, H McShane, M Parkes, D Peckham, P Pfeffer, A Sayer, A Sheikh, A A R Thompson, N Williams and core management group representation

Executive Board

Chair C E Brightling, representation from the core management group, each working group and platforms

Platforms

Bioresource

W Greenhalf (*Co-Lead*), M G Semple (*Co-Lead*), M Ashworth, H E Hardwick, L Lavelle-Langham, W Reynolds, M Sereno, R M Saunders, A Singapuri, V Shaw, A Shikotra, B Venson, L V Wain

Data Hub

A B Docherty (*Co-Lead*), E M Harrison (*Co-Lead*), A Sheikh (*Co-Lead*), J K Baillie, C E Brightling, L Daines, R Free, R A Evans, S Kerr, O C Leavy, N I Lone, D Lozano-Rojas, H J C McAuley, K Ntotsis, R Pius, J K Quint, M Richardson, M Sereno, M Thorpe, L V Wain

Imaging Alliance

M Halling-Brown (*Co-Lead*), F Gleeson (*Co-Lead*), J Jacob (*Co-Lead*), S Neubauer (*Co-Lead*) B Raman (*Co-Lead*) S Siddiqui (*Co-Lead*) J M Wild (*Co-Lead*), S Aslani, G Baxter, M Beggs, C Bloomfield, M P Cassar, A Chiribiri, E Cox, D J Cuthbertson, M Halling-Brown, V M Ferreira, L Finnigan, S Francis, P Jezzard, G J Kemp, H Lamlum, E Lukaschuk, C Manisty, G P McCann , C McCracken, K McGlynn , R Menke , C A Miller , A J Moss, T E Nichols, C Nikolaidou , C O'Brien , G Ogbole, B Rangelov, D P O'Regan , A Pakzad, S Piechnik , S Plein, I Propescu, A A Samat, L Saunders, Z B Sanders, R Steeds, T Treibel, E M Tunnicliffe, M Webster, J Willoughby, J Weir McCall, C Xie, M Xu

Omics

L V Wain (*Co-Lead),* J K Baillie (*Co-Lead*), H Baxendale, C E Brightling, M Brown, J D Chalmers, R A Evans, B Gooptu, W Greenhalf, H E Hardwick, R G Jenkins, D Jones, I Koychev, C Langenberg, A Lawrie, P L Molyneaux, A Shikotra, J Pearl, M Ralser, N Sattar, R M Saunders, J T Scott, T Shaw, D Thomas, D Wilkinson

Working Groups

Airways

L G Heaney (*Co-Lead*), A De Soyza (*Co-Lead*), D Adeloye, C E Brightling, J S Brown, J Busby, J D Chalmers, C Echevarria, L Daines, O Elneima, RA Evans, J R Hurst, P Novotny, C Nicolaou, P Pfeffer, K Poinasamy, J K Quint, I Rudan, E Sapey, M Shankar-Hari, A Sheikh, S Siddiqui, S Walker, B Zheng

Brain

J R Geddes (*Lead*), M Hotopf *(Co-Lead),* K Abel, R Ahmed, L Allan, C Armour, D Baguley, D Baldwin, C Ballard, K Bhui, G Breen, K Breeze, M Broome, T Brugha, E Bullmore, D Burn, F Callard, J Cavanagh, T Chalder, D Clark, A David, B Deakin, H Dobson, B Elliott, J Evans, RA Evans, R Francis, E Guthrie, P Harrison, M Henderson, A Hosseini, N Huneke, M Husain, T Jackson, I Jones, T Kabir, P Kitterick, A Korszun, I Koychev, J Kwan, A Lingford-Hughes, P Mansoori, H McAllister-Williams, K McIvor, B Michael, L Milligan, R Morriss, E Mukaetova-Ladinska, K Munro, A Nevado-Holgado, T Nicholson, C Nicolaou, S Paddick, C Pariante, J Pimm, K Saunders, M Sharpe, G Simons, J P Taylor, R Upthegrove, S Wessely

Cardiac

G P McCann (*Lead*), S Amoils, C Antoniades, A Banerjee, A Bularga, C Berry, P Chowienczyk, J P Greenwood, A D Hughes, K Khunti, C Lawson, N L Mills, A J Moss, S Neubauer, B Raman, A N Sattar, C L Sudlow, M Toshner,

Immunology

P J M Openshaw (*Lead*), D Altmann, J K Baillie, R Batterham, H Baxendale, N Bishop, C E Brightling, P C Calder, C M Efstathiou, R A Evans, J L Heeney, T Hussell, P Klenerman, F Liew, J M Lord, P Moss, S L Rowland-Jones, W Schwaeble, M G Semple, R S Thwaites, L Turtle, L V Wain, S Walmsley, D Wraith

Intensive Care

M J Rowland (*Lead*), A Rostron (*Co-Lead*), J K Baillie, B Connolly, A B Docherty, N I Lone, D F McAuley, D Parekh, A Rostron, J Simpson, C Summers

Lung Fibrosis

R G Jenkins (*Co-Lead*), J Porter (*Co-Lead*), R J Allen, R Aul, J K Baillie, S Barratt, P Beirne, J Blaikley, R C Chambers, N Chaudhuri, C Coleman, E Denneny, L Fabbri, P M George, M Gibbons, F Gleeson, B Gooptu, B Guillen Guio, I Hall, N A Hanley, L P Ho, E Hufton, J Jacob, I Jarrold, G Jenkins, S Johnson, M G Jones, S Jones, F Khan, P Mehta, J Mitchell, P L Molyneaux, J E Pearl, K Piper Hanley, K Poinasamy, J Quint, D Parekh, P Rivera-Ortega, L C Saunders, M G Semple, J Simpson, D Smith, M Spears, L G Spencer, S Stanel, I Stewart, A A R Thompson, D Thickett, R Thwaites, L V Wain, S Walker, S Walsh, J M Wild, D G Wootton, L Wright

Metabolic

S Heller (*Co-Lead*), M J Davies (*Co-Lead*), H Atkins, S Bain, J Dennis, K Ismail, D Johnston, P Kar, K Khunti, C Langenberg, P McArdle, A McGovern, T Peto, J Petrie, E Robertson, N Sattar, K Shah, J Valabhji, B Young

Pulmonary and Systematic Vasculature

L S Howard (*Co-Lead*), Mark Toshner (*Co-Lead*), C Berry, P Chowienczyk, A Lawrie, O C Leavy, J Mitchell, J Newman, L Price, J Quint, A Reddy, J Rossdale, N Sattar, C Sudlow, A A R Thompson, J M Wild, M Wilkins

Rehabilitation, Sarcopenia and Fatigue

S J Singh (*Co-Lead*), W D-C Man (*Co-Lead*), J M Lord (*Co-Lead*), N J Greening (*Co-Lead*), T Chalder (*Co-Lead*), J T Scott (*Co-Lead*), N Armstrong, E Baldry, M Baldwin, N Basu, M Beadsworth, L Bishop, C E Bolton, A Briggs, M Buch, G Carson, J Cavanagh, H Chinoy, C Dawson, E Daynes, S Defres, R A Evans, L Gardiner, P Greenhaff, S Greenwood, M Harvie, L HOuchen-Wolloff, M Husain, S MacDonald, A McArdle, H J C McAuley, A McMahon, M McNarry, G Mills, C Nolan, K O'Donnell, D Parekh, Pimm, J Sargent, L Sigfrid, M Steiner, D Stensel, A L Tan, I Vogiatzis, J Whitney, D Wilkinson, D Wilson, M Witham, D G Wootton, T Yates

Renal

D Thomas (*Lead*), N Brunskill (*Co-Lead*), S Francis (*Co-Lead*), S Greenwood (*Co-Lead*), C Laing (*Co-Lead*), K Bramham, P Chowdhury, A Frankel, L Lightstone, S McAdoo, K McCafferty, M Ostermann, N Selby, C Sharpe, M Willicombe

Patient Public Engagement Group

L Houchen-Wolloff (*Lead*), J Bunker, R Gill, C Hastie, R Nathu, N Rogers, N Smith

Local Clinical Centre PHOSP-COVID trial staff

(listed in alphabetical order)

Airedale NHS Foundation Trust

A Shaw (PI), L Armstrong, B Hairsine, H Henson, C Kurasz, L Shenton

Aneurin Bevan University Health Board

S Fairbairn (PI), A Dell, N Hawkings, J Haworth, M Hoare, A Lucey, V Lewis, G Mallison, H Nassa, C Pennington, A Price, C Price, A Storrie, G Willis, S Young

Barts Health NHS Trust & Queen Mary University of London

P Pfeffer (PI), K Chong-James, C David, W Y James, C Manisty, A Martineau, O Zongo

Barnsley Hospital NHS Foundation Trust

A Sanderson (PI)

Belfast Health and Social Care Trust & Queen's University Belfast

L G Heaney (PI), C Armour, V Brown, T Craig, S Drain, B King, N Magee, D McAulay, E Major, L McGarvey, J McGinness, R Stone

Betsi Cadwaladr University Health Board

A Haggar (PI), A Bolger, F Davies, J Lewis, A Lloyd, R Manley, E McIvor, D Menzies, K Roberts, W Saxon, D Southern, C Subbe, V Whitehead

Borders General Hospital, NHS Borders

H El-Taweel (PI), J Dawson, L Robinson

Bradford Teaching Hospitals NHS Foundation Trust

D Saralaya (PI), L Brear, K Regan, K Storton

Cambridge University Hospitals NHS Foundation Trust, NIHR Cambridge Clinical Research Facility & University of Cambridge

J Fuld (PI), A Bermperi, I Cruz, K Dempsey, A Elmer, H Jones, S Jose, S Marciniak, M Parkes, C Ribeiro, J Taylor, M Toshner, L Watson, J Weir McCall, J Worsley

Cardiff and Vale University Health Board

R Sabit (PI), L Broad, A Buttress, T Evans, M Haynes, L Jones, L Knibbs, A McQueen, C Oliver, K Paradowski, J Williams

Chesterfield Royal Hospital NHS Trust

E Harris (PI), C Sampson

Cwm Taf Morgannwg University Health Board

C Lynch (PI), E Davies, C Evenden , A Hancock, K Hancock, M Rees , L Roche, N Stroud, T Thomas-Woods

East Cheshire NHS Trust

M Babores (PI), J Bradley-Potts, M Holland, N Keenan, S Shashaa, H Wassall

East Kent Hospitals University NHS Foundation Trust

E Beranova (PI), H Weston (PI), T Cosier, L Austin, J Deery, T Hazelton, C Price, H Ramos, R Solly, S Turney

Gateshead NHS Trust

L Pearce (PI), W McCormick, S Pugmire, W Stoker, A Wilson

Guy's and St Thomas' NHS Foundation Trust

N Hart (PI), LA Aguilar Jimenez, G Arbane, S Betts, K Bisnauthsing, A Dewar, P Chowdhury, A Chiribiri, A Dewar, G Kaltsakas, H Kerslake, MM Magtoto, P Marino, LM Martinez, C O'Brien, M Ostermann, J Rossdale, TS Solano, E Wynn

Hampshire Hospitals NHS Foundation Trust

N Williams (PI), W Storrar (PI), M Alvarez Corral, A Arias, E Bevan, D Griffin, J Martin, J Owen,

S Payne, A Prabhu, A Reed, C Wrey Brown

Harrogate and District NHD Foundation Trust

C Lawson (PI), T Burdett, J Featherstone, A Layton, C Mills, L Stephenson,

Hull University Teaching Hospitals NHS Trust & University of Hull

N Easom (PI), P Atkin, K Brindle, M G Crooks, K Drury, R Flockton, L Holdsworth, A Richards, D L Sykes, S Thackray-Nocera, C Wright

Hywel Dda University Health Board

K E Lewis (PI), A Mohamed (PI), G Ross (PI), S Coetzee, K Davies, R Hughes, R Loosley, L O'Brien, Z Omar, H McGuinness, E Perkins, J Phipps, A Taylor, H Tench, R Wolf-Roberts

Imperial College Healthcare NHS Trust & Imperial College London

L S Howard (PI), O Kon (PI), D C Thomas (PI), S Anifowose, L Burden, E Calvelo, B Card, C Carr, E R Chilvers, D Copeland, P Cullinan, P Daly, C M Efstathiou, L Evison, T Fayzan, H Gordon, S Haq, R G Jenkins, C King, F Liew, K March, M Mariveles, L McLeavey, N Mohamed, S Moriera, U Munawar, J Nunag, U Nwanguma, L Orriss- Dib, D P O'Regan, A Ross, M Roy, E Russell, K Samuel, J Schronce, N Simpson, L Tarusan, C Wood, N Yasmin

Kettering General Hospital NHS Trust

R Reddy (PI), A-M Guerdette, M Hewitt, K Warwick, S White

King's College Hospital NHS Foundation Trust & Kings College London

A M Shah (PI), C J Jolley (PI), O Adeyemi, R Adrego, H Assefa-Kebede, J Breeze, M Brown, S Byrne, T Chalder, A Chiribiri, P Dulawan, N Hart, A Hayday, A Hoare, A Knighton, M Malim, C O'Brien, S Patale, I Peralta, N Powell, A Ramos, K Shevket, F Speranza, A Te

Leeds Teaching Hospitals & University of Leeds

P Beirne (PI), A Ashworth, J Clarke, C Coupland, M Dalton, E Wade, C Favager, J Greenwood, J Glossop, L Hall, T Hardy, A Humphries, J Murira, D Peckham, S Plein, J Rangeley, G Saalmink, A L Tan, B Whittam, N Window, J Woods,

Lewisham & Greenwich NHS Trust

G Coakley (PI)

Liverpool University Hospitals NHS Foundation Trust & University of Liverpool

D G Wootton (PI), L Turtle (PI), L Allerton, AM All, M Beadsworth, A Berridge, J Brown, S Cooper, A Cross, D J Cuthbertson, S Defres, S L Dobson, J Earley, N French, W Greenhalf, H E Hardwick, K Hainey, J Hawkes, V Highett, S Kaprowska, G J Kemp, AL Key, S Koprowska, L Lavelle-Langham, N Lewis-Burke, G Madzamba, F Malein, S Marsh, C Mears, L Melling, M J Noonan, L Poll, J Pratt, E Richardson, A Rowe, M G Semple, V Shaw, K A Tripp, B Vinson, L O Wajero, S A Williams-Howard, J Wyles

London North West University Healthcare NHS Trust

S N Diwanji (PI), P Papineni (PI), S Gurram, S Quaid, G F Tiongson, E Watson

Manchester University NHS Foundation Trust & University of Manchester

B Al-Sheklly (PI), A Horsley (PI), C Avram, P Barran, J Blaikely, M Buch, N Choudhury, D Faluyi, T Felton, T Gorsuch, N A Hanley, T Hussell, Z Kausar, C A Miller, N Odell, R Osbourne, K Piper Hanley, K Radhakrishnan, S Stockdale, D Trivedi

Newcastle upon Tyne Hospitals NHS Foundation Trust & University of Newcastle

A De Soyza (PI), C Echevarria (PI), A Ayoub, J Brown, G Burns, G Davies, H Fisher, C Francis, A Greenhalgh, P Hogarth, J Hughes, K Jiwa, G Jones, G MacGowan, D Price, A Sayer, J Simpson, H Tedd, S Thomas, S West, M Witham, S Wright, A Young

NHS Dumfries and Galloway

M J McMahon (PI), P Neill

NHS Greater Glasgow and Clyde Health Board & University of Glasgow

D Anderson (PI), H Bayes (PI), C Berry (PI), D Grieve (PI), I B McInnes (PI), N Basu, A Brown, A Dougherty, K Fallon, L Gilmour, K Mangion, A Morrow, K Scott, R Sykes, R Touyz

NHS Highland

E K Sage (PI), F Barrett, A Donaldson

NHS Lanarkshire

M Patel (PI), D Bell, A Brown, M Brown, R Hamil, K Leitch, L Macliver, J Quigley, A Smith, B Welsh

NHS Lothian & University of Edinburgh

G Choudhury (PI), J K Baillie, S Clohisey, A Deans, A B Docherty, J Furniss, E M Harrison, S Kelly, N I Lone, D E Newby, A Sheikh

NHS Tayside & University of Dundee

J D Chalmers (PI), D Connell, A Elliott, C Deas, J George, S Mohammed, J Rowland, A R Solstice, D Sutherland, C J Tee

North Bristol NHS Trust & University of Bristol

N Maskell (PI), D Arnold, S Barrett, H Adamali, A Dipper, S Dunn, A Morley, L Morrison, L Stadon, S Waterson, H Welch

North Middlesex Hospital NHS Trust

B Jayaraman (PI), T Light

Nottingham University Hospitals NHS Trust & University of Nottingham

C E Bolton (PI), P Almeida, J Bonnington, M Chrystal, E Cox, C Dupont, S Francis, P Greenhaff, A Gupta, L Howard, W Jang, S Linford, L Matthews, R Needham, A Nikolaidis, S Prosper, K Shaw, A K Thomas

Oxford University Hospitals NHS Foundation Trust & University of Oxford

L P Ho (PI), N M Rahman (PI), M Ainsworth, A Alamoudi, M Beggs, A Bates, A Bloss, A Burns, P Carter, M Cassar, K M Channon, J Chen, F Conneh, T Dong, R I Evans, E Fraser, X Fu, J R Geddes, F Gleeson, P Harrison, M Havinden-Williams, P Jezzard, N Kanellakis, I Koychev, P Kurupati, X Li, E Lukaschuk, K McGlynn, H McShane, C

Megson, K Motohashi, S Neubauer, D Nicoll, G Ogg, E Pacpaco, M Pavlides, Y Peng, N Petousi, J Propescu, N Rahman, B Raman, M J Rowland, K Saunders, M Sharpe, N Talbot, E Tunnicliffe

Royal Brompton and Harefield Clinical Group, Guy's and St Thomas' NHS Foundation Trust.

W D-C Man (PI), B Patel (PI), R E Barker, D Cristiano, N Dormand, M Gummadi, S Kon, K Liyanage, C M Nolan, S Patel, O Polgar, P Shah, S J Singh, J A Walsh

Royal Free London NHS Foundation Trust

J Hurst (PI), H Jarvis (PI), S Mandal (PI), S Ahmad, S Brill, L Lim, D Matila, O Olaosebikan, C Singh

Royal Papworth Hospital NHS Foundation Trust

M Toshner (PI), H Baxendale, L Garner, C Johnson, J Mackie, A Michael, J Pack, K Paques, H Parfrey, J Parmar

Salford Royal NHS Foundation Trust

N Diar Bakerly (PI), P Dark, D Evans, E Hardy, A Harvey, D Holgate, S Knight, N Mairs, N Majeed, L McMorrow, J Oxton, J Pendlebury, C Summersgill, R Ugwuoke, S Whittaker

Salisbury NHS Foundation Trust

W Matimba-Mupaya (PI), S Strong-Sheldrake

Sheffield Teaching NHS Foundation Trust & University of Sheffield

S L Rowland-Jones (PI), A A R Thompson (Co PI), J Bagshaw, M Begum, K Birchall, R Butcher, H Carborn, F Chan, K Chapman, Y Cheng, L Chetham, C Clark, Z Coburn, J Cole, M Dixon, A Fairman, J Finnigan, L Finnigan, H Foot, D Foote, A Ford, R Gregory, K Harrington, L Haslam, L Hesselden, J Hockridge, A Holbourn, B Holroyd-Hind, L Holt, A Howell, E Hurditch, F Ilyas, C Jarman, A Lawrie, E Lee, J-H Lee, R Lenagh, A Lye, I Macharia, M Marshall, A Mbuyisa, J McNeill, S Megson, J Meiring, L Milner, S Misra, H Newell, T Newman, C Norman, L Nwafor, D Pattenadk, M Plowright, J Porter, P Ravencroft, C Roddis, J Rodger, P Saunders, J Sidebottom, J Smith, L Smith, N Steele, G Stephens, R Stimpson, B Thamu, N Tinker, K Turner, H Turton, P Wade, S Walker, J Watson, J M Wild, I Wilson, A Zawia

St George's University Hospitals NHS Foundation Trust

R Aul (PI), M Ali, A Dunleavy (PI), D Forton, N Msimanga, M Mencias, T Samakomva, S Siddique, J Teixeira, V Tavoukjian

Sherwood Forest Hospitals NHS Foundation Trust

J Hutchinson (PI), L Allsop, K Bennett, P Buckley, M Flynn, M Gill, C Goodwin, M Greatorex, H Gregory, C Heeley, L Holloway, M Holmes, J Kirk, W Lovegrove, TA Sewell, S Shelton, D Sissons, K Slack, S Smith, D Sowter, S Turner, V Whitworth, I Wynter

Shropshire Community Health NHS Trust

L Warburton (PI), S Painter, J Tomlinson

Somerset NHS Foundation Trust

C Vickers (PI), T Wainwright, D Redwood, J Tilley, S Palmer

Swansea Bay University Health Board

G A Davies (PI), L Connor, A Cook, T Rees, F Thaivalappil, C Thomas

Tameside and Glossop Integrated Care NHS Foundation

A Butt (PI), M Coulding, H Jones, S Kilroy, J McCormick, J McIntosh, H Savill, V Turner, J Vere

The Great Western Hospital Foundation Trust

E Fraile (PI), J Ugoji

The Hillingdon Hospitals NHS Foundation Trust

S S Kon (PI), H Lota, G Landers, M Nasseri, S Portukhay

The Rotherham NHS Foundation Trust

A Hormis (PI), A Daniels, J Ingham, L Zeidan

United Lincolnshire Hospitals NHS Trust

M Chablani (PI), L Osborne

University College London Hospital & University College London

M Marks (PI), J S Brown (PI), N Ahwireng, B Bang, D Basire, R C Chambers, A Checkley, R Evans, M Heightman, T Hillman, J Hurst, J Jacob, S Janes, R Jastrub, M Lipman, S Logan, D Lomas, M Merida Morillas, A Pakzad, H Plant, J C Porter, K Roy, E Wall, B Williams, M Xu

University Hospital Birmingham NHS Foundation Trust & University of Birmingham

D Parekh (PI), N Ahmad Haider, C Atkin, R Baggott, M Bates, A Botkai, A Casey, B Cooper, J Dasgin, K Draxlbauer, N Gautam, J Hazeldine, T Hiwot, S Holden, K Isaacs, T Jackson, S Johnson, V Kamwa, D Lewis, J M Lord, S Madathil, C McGhee, K Mcgee, A Neal, A Newton Cox, J Nyaboko, D Parekh, Z Peterkin, H Qureshi, B Rangelov, L Ratcliffe, E Sapey, J Short, T Soulsby, R Steeds, J Stockley, Z Suleiman, T Thompson, M Ventura, S Walder, C Welch, D Wilson, S Yasmin, K P Yip

University Hospitals of Derby and Burton

P Beckett (PI) C Dickens, U Nanda

University Hospitals of Leicester NHS Trust & University of Leicester

C E Brightling (CI), R A Evans (PI), M Aljaroof, N Armstrong, H Arnold, H Aung, M Bakali, M Bakau, M Baldwin, M Bingham, M Bourne, C Bourne, N Brunskill, P Cairns, L Carr, A Charalambou, C Christie, M J Davies, S Diver, S Edwards, C Edwardson, O Elneima, H Evans, J Finch, S Glover, N Goodman, B Gootpu, N J Greening, K Hadley, P Haldar, B Hargadon, V C Harris, L Houchen-Wolloff, W Ibrahim, L Ingram, K Khunti, A Lea, D Lee, D Lozano-Rojas, G P McCann, H J C McAuley, P McCourt, T Mcnally, G Mills, A Moss, W Monteiro, K Ntotsis, M Pareek, S Parker, A Rowland, A Prickett, I N Qureshi, R J Russell, N Samani, M Sereno, M Sharma, A Shikotra, S Siddiqui, A Singapuri, S J Singh, J Skeemer, M Soares, E Stringer, T Thornton, M Tobin, E Turner, L V Wain, T J C Ward, F Woodhead, J Wormleighton, T Yates, A Yousuf,

University Hospital Southampton NHS Foundation Trust & University of Southampton

M G Jones (PI), C Childs, R Djukanovic, S Fletcher, M Harvey, E Marouzet, B Marshall, R Samuel, T Sass, T Wallis, H Wheeler

Whittington Health NHS

R Dharmagunawardena (PI), E Bright, P Crisp, M Stern

Wirral University Teaching Hospital

A Wight (PI), L Bailey, A Reddington

Wrightington Wigan and Leigh NHS trust

A Ashish (PI), J Cooper, E Robinson

Yeovil District Hospital NHS Foundation Trust

A Broadley (PI)

York & Scarborough NHS Foundation Trust

K Howard (PI), L Barman, C Brookes, K Elliott. L Griffiths, Z Guy, D Ionita, H Redfearn, C Sarginson

A Turnbull

Health and Care Research Wales

Y Ellis

London School of Hygiene & Tropical Medicine (LSHTM)

M Marks, A Briggs

NIHR Office for Clinical Research Infrastructure

K Holmes

Patient Public Involvement Leads

Asthma UK and British Lung Foundation Partnership - K Poinasamy, S Walker

Royal Surrey NHS Foundation Trust

M Halling-Brown

South London and Maudsley NHS Foundation Trust & Kings College London

G Breen, M Hotopf

Swansea University & Swansea Welsh Network

K Lewis, N Williams

References

1. Office for National Statistics. National Statistics Postcode Lookup (February 2020). February 02, 2020. https://geoportal.statistics.gov.uk/datasets/national-statistics-postcode-lookup-february2020 (accessed December 01, 2022).
2. WHO Working Group on the Clinical Characterisation and Management of COVID-19 infection. "A minimal common outcome measure set for COVID-19 clinical research." *The Lancet. Infectious diseases* 2020; **20**(8): e192-e197.
3. Johnson SU, Ulvenes PG, Øktedalen T, Hoffart A. Psychometric properties of the general anxiety disorder 7-item (GAD-7) scale in a heterogeneous psychiatric sample. *Front Psychol* 2019; **10**:1713.
4. Levis B, Benedetti A, Thombs BD. Accuracy of Patient Health Questionnaire-9 (PHQ-9) for screening to detect major depression: individual participant data meta-analysis. *BMJ* 2019; **365**:l1476.
5. Contractor AA, Elhai JD, Fine TH, et al. Latent profile analyses of posttraumatic stress disorder, depression and generalized anxiety disorder symptoms in trauma-exposed soldiers. *J Psychiatr Res* 2015; **68**: 19-26.
6. Weathers FWL, B.T. Keane, T.M. Palmieri, P.A. Marx, B.P. & Schnurr, P.P. The PTSD Checklist for DSM-5 (PCL-5). 2013. www.ptsd.va.gov (accessed May 30, 2023).
7. Yorke J, Moosavi SH, Shuldham C, Jones PW. Quantification of dyspnoea using descriptors: development and initial testing of the Dyspnoea-12. *Thorax* 2010; **65**(1): 21-6.
8. FACIT.org. Functional Assessment of Chronic Illness Therapy – Fatigue: A 13-item FACIT Fatigue Scale. https://www.facit.org/measures/FACIT-F (accessed May 30, 2023).
9. Butt Z, Lai JS, Rao D, Heinemann AW, Bill A, Cella D. Measurement of fatigue in cancer, stroke, and HIV using the Functional Assessment of Chronic Illness Therapy - Fatigue (FACIT-F) scale. *J Psychosom Res* 2013; **74**(1): 64-8.
10. Vasunilashorn S, Coppin AK, Patel KV, et al. Use of the Short Physical Performance Battery Score to predict loss of ability to walk 400 meters: analysis from the InCHIANTI study. *J Gerontol A Biol Sci Med Sci* 2009; **64**(2): 223-9.
11. Guralnik JM, Simonsick EM, Ferrucci L, et al. A short physical performance battery assessing lower extremity function: association with self-reported disability and prediction of mortality and nursing home admission. *J Gerontol* 1994; **49**(2): M85-M94.
12. Fish J. Short Physical Performance Battery. In: Kreutzer JS, DeLuca J, Caplan B, eds. Encyclopedia of Clinical Neuropsychology. New York, NY: Springer New York; 2011: 2289-91.

13. Singh SJ, Morgan M, Scott S, Walters D, Hardman AE. Development of a shuttle walking test of disability in patients with chronic airways obstruction. *Thorax* 1992; **47**(12): 1019-24.
14. Probst VS, Hernandes NA, Teixeira DC, et al. Reference values for the incremental shuttle walking test. *Respir Med* 2012; **106**(2): 243-8.
15. Rockwood K, Song X, MacKnight C, et al. A global clinical measure of fitness and frailty in elderly people. *CMAJ* 2005; **173**(5): 489-95.
16. Nasreddine ZS. MoCA Montreal Cognitive Assessment Training & Certification. https://www.mocatest.org/training-certification/ (accessed May 30, 2023).
17. Carson N, Leach L, Murphy KJ. A re-examination of Montreal Cognitive Assessment (MoCA) cutoff scores. *Int J Geriatr Psychiatry* 2018; **33**(2): 379-88.
18. Graham BL, Steenbruggen I, Miller MR, et al. Standardization of spirometry 2019 update. An official American thoracic Society and European respiratory Society technical statement. *Am J Respir Crit Care Med* 2019; **200**(8): e70-e88.
19. Quanjer PH, Stanojevic S, Cole TJ, et al. Multi-ethnic reference values for spirometry for the 3–95-yr age range: the global lung function 2012 equations. Eur Respiratory Soc; 2012. 40
20. Stanojevic S, Graham BL, Cooper BG, et al. Official ERS technical standards: Global Lung Function Initiative reference values for the carbon monoxide transfer factor for Caucasians (vol 50, 1700010, 2017). *Eur Respir J* 2020; **56**(4).
21. Stanojevic S, Graham BL, Cooper BG, et al. Official ERS technical standards: Global Lung Function Initiative reference values for the carbon monoxide transfer factor for Caucasians. *Eur Respir J* 2017; **50**(3).
22. Vestbo J, Hurd SS, Agustí AG, et al. Global strategy for the diagnosis, management, and prevention of chronic obstructive pulmonary disease: GOLD executive summary. *Am J Respir Crit Care Med* 2013; **187**(4): 347-65.
23. Acute heart failure: diagnosis and management. National Institute for Health and Care Excellence; 2014.
24. Chronic heart failure in adults: diagnosis and management. National Institute for Health and Care Excellence; 2018.
25. International Expert Committee Report on the Role of the A1C Assay in the Diagnosis of Diabetes. *Diabetes Care* 2009; **32**(7): 1327-34.
26. Evans RA, McAuley H, Harrison EM, et al. Physical, cognitive, and mental health impacts of COVID-19 after hospitalisation (PHOSP-COVID): a UK multicentre, prospective cohort study. Lancet Respir Med. 2021;9(11):1275-1287
27. Gerlinger C, Bamber L, Leverkus F, et al. Comparing the EQ-5D-5L utility index based on value sets of different countries: impact on the interpretation of clinical study results. *BMC Res Notes* 2019; **12**(1): 1-6.
28. Herdman M, Gudex C, Lloyd A, et al. Development and preliminary testing of the new five level version of EQ-5D (EQ-5D-5L). *Qual Life Res* 2011; **20**(10): 1727-36.
29. Creating Disability Severity Indicators Using the WG Short Set on Functioning (WG-SS) (CSPro). January 2021. https://www.washingtongroup-disability.com/fileadmin/uploads/wg/WG_Document__5H_-_Analytic_Guidelines_for_the_WG-SS__Severity_Indicators_-_CSPro_.pdf (accessed May 30, 2023).
